# Single-cell transcriptomics of the ocular anterior segment: a comprehensive review

**DOI:** 10.1038/s41433-023-02539-3

**Published:** 2023-05-03

**Authors:** Sofia Ahsanuddin, Albert Y. Wu

**Affiliations:** 1 Department of Ophthalmology, Byers Eye Institute, Stanford University School of Medicine, Stanford, CA USA; 2 Department of Ophthalmology, New York Eye and Ear Infirmary of Mount Sinai, New York City, NY USA; 3 Department of Ophthalmology, Icahn School of Medicine at Mount Sinai, New York City, NY USA

**Keywords:** Gene expression, Mechanisms of disease, Conjunctival diseases, Corneal diseases, Inflammation

## Abstract

Elucidating the cellular and genetic composition of ocular tissues is essential for uncovering the pathophysiology of ocular diseases. Since the introduction of single-cell RNA sequencing (scRNA-seq) in 2009, vision researchers have performed extensive single-cell analyses to better understand transcriptome complexity and heterogeneity of ocular structures. This technology has revolutionized our ability to identify rare cell populations and to make cross-species comparisons of gene expression in both steady state and disease conditions. Importantly, single-cell transcriptomic analyses have enabled the identification of cell-type specific gene markers and signalling pathways between ocular cell populations. While most scRNA-seq studies have been conducted on retinal tissues, large-scale transcriptomic atlases pertaining to the ocular anterior segment have also been constructed in the past three years. This timely review provides vision researchers with an overview of scRNA-seq experimental design, technical limitations, and clinical applications in a variety of anterior segment-related ocular pathologies. We review open-access anterior segment-related scRNA-seq datasets and illustrate how scRNA-seq can be an indispensable tool for the development of targeted therapeutics.

## Abstract (zh)

阐明眼组织细胞和基因的组成对于明确眼病的病理生理学是至关重要的。自从2009年单细胞RNA测序 (scRNA-seq) 引进以来, 视觉研究人员为了来更好理解眼部结构转录组的复杂性和异质性, 进行了深入的单细胞组学研究。这项技术彻底改变了我们辨别稀有细胞群及在稳态和病态条件下对基因表达进行跨物种比较的能力。重要的是, 单细胞转录组分析使我们能在眼细胞群之间识别细胞类型的特殊的基因标记物以及信号通路。虽然大部分scRNA-seq用于视网膜组织研究, 但在过去三年中也设计了大规模眼前节转录组图谱。这篇适时的综述为视觉研究人员针对不同眼前节相关眼病提供了scRNA-seq实验设计、技术限制和临床应用的概述。我们回顾了眼前节相关scRNA-scq的公开的公开数据集, 并解释scRNA-seq为何是靶向治疗不可或缺的工具。

## Introduction

Technological advances in mRNA phenotyping methods have enabled unprecedented access to transcriptomic data in ophthalmology [1]. As a proxy to the proteome, the “transcriptome” offers insight into how gene expression correlates with cellular physiology and function, thereby revealing previously unknown cellular dynamics in ensembles of large cell numbers [2, 3]. Prior to transcriptomics, the earliest applications of immunohistochemistry in ophthalmic pathology enabled researchers to define cellular phenotype by protein expression, while in-situ hybridization enabled precise spatial RNA sequence localization in a select tissue sample [4]. To construct gene expression profiles at different time-points, researchers harnessed subtractive hybridization to create complementary DNA (cDNA) libraries of various ocular structures such as retina, retinal pigment epithelial (RPE), and ciliary epithelium [5–8]. This technique has been demonstrated to be effective for investigating gene expression in complex disease states such as uveal melanoma [9].

Expression studies involving differential display, expressed sequence tags (EST), serial analysis of gene expression (SAGE), and hybridization-based microarray technologies have similarly facilitated differentially expressed gene identification in ophthalmology [10]. Eventually, the development of next-generation sequencing (NGS) techniques like RNA-sequencing (RNA-Seq) pooled large cell numbers and allowed researchers to generate more comprehensive cell atlases comprised of both known and unknown mRNA transcripts, a feat that was previously limited to known transcripts [11, 12]. The high throughput of this technology allowed for novel gene marker detection and provided insight into alternative splicing as an important regulatory factor of cellular heterogeneity and pathogenesis in many disease states [13]. Nonetheless, all of these technologies involve analysis of large numbers of aggregated cells, which hinders more granular assessments of gene expression dynamics in highly heterogenous tissues like those found in the eye. Because it lacks individual cell-type resolution, pooled RNA-seq analyses also cannot provide insight into each cell type’s relative abundance and how gene expression differs by cell type within a sample [14].

Since the first single-cell RNA sequencing (scRNA-seq) study was described by Tang et al. in 2009, the technology has generated unprecedented levels of interest in the vision research community [3, 15]. It has proven to be a versatile and powerful tool for understanding the transcriptional heterogeneity of ocular tissues at single-cell resolution. ScRNA-seq studies have rapidly expanded from profiling eight cells to 1.3 million cells [3]. In comparison to bulk RNA-seq, scRNA-seq enables the identification of rare cell populations, stem cells, novel biomarkers, lineage tracing between various cellular states, and transcriptional responses to drug therapy over time, all of which allow researchers to understand the molecular mechanisms driving disease processes at high-resolution [16]. Importantly, scRNA-seq has been demonstrated to be useful for developing targeted therapeutic interventions in highly heterogenous tissues because it enables investigations of cell type, monoallelic gene expression, gene co-expression, splicing patterns, and gene regulatory networks in a parallelized manner.

In the vision research community, scRNA-seq has primarily been utilized to elucidate the different cell types in the retina, RPE, and choroid of mice, primates, humans, and induced pluripotent stem cell (iPSC)-derived organoids [1, 17]. More recently, however, the technology has been utilized to elucidate the normal development and physiology of the ocular anterior segment, which consists of the cornea, conjunctiva, iris, ciliary body, crystalline lens, trabecular meshwork (TM), Schlemm canal (SC), and ciliary muscle [18–25]. These studies have made unparalleled contributions to our understanding of limbal epithelial stem cell populations and their role in mediating corneal and conjunctival homoeostasis and wound healing. Additionally, scRNA-seq studies like Dou et al.’s study have provided important insights into embryonic development, stem cell differentiation, cell type-specific transcription factors, and signalling pathways involved in disease states such as keratoconus, glaucoma, uveal melanoma, and Fuchs endothelial corneal dystrophy (FECD) [26].

The unprecedented interrogation of ocular transcriptomic data has enormous potential for informing targeted therapeutics of complex ocular diseases. In this review, we describe experimental considerations and clinical applications for scRNA-seq pertaining to the ocular anterior segment. A succinct scRNA-seq terminology glossary is included in Table 1. We also describe potential biases and technical limitations of scRNA-seq data and discuss advantages of conducting single-nuclei RNA-seq (snRNA-seq) in ophthalmology. Equipped with this knowledge, one can better harness scRNA and snRNA-seq data to assess the molecular mechanisms underlying anterior segment development and disease [27]. Future integration of scRNA-seq data with clinical data can enable the identification of previously unknown biomarkers, thereby empowering clinicians to develop novel therapies that overcome treatment resistance and improve prognosis.

**Table 1:** Glossary of scRNA-seq terminology.

Term	Definition
Coverage	Number of sequencing reads that align to a reference genome
Batch effects	Non-biological variation in gene expression attributed to technical or systematic biases that occur during the handling of distinct batches of samples
Doublets	Two or more cells captured in the same droplet or bead
Amplification bias	Amplification of certain genes over others
Sequencing Depth	A measurement of the sequencing capacity defined as the number of raw reads per cell. Transcript with lower abundance are identified by sequencing deeper
Sensitivity	Ability to detect gene expression for a given sequencing depth
Accuracy	How accurate the expression value of a gene is in comparison to others within a cell based on spike-in experiments
Spike-in experiment	An experiment where a molecule(s) is introduced to a sample in order to calibrate detection of that molecule and account for technical noise and biases
Unique Molecular Identifiers (UMIs)	RNA molecules are barcoded with random oligonucleotides of varying lengths called UMIs to result in uniquely tagged molecules
Sequencing library	Amplified complementary DNA (cDNA) for a single cell

## Methods

In September 2022, PubMed, Google Scholar, and NCBI GEO databases were systematically queried for the terms ‘single cell RNA sequencing eye,’ ‘scRNA-seq eye,’ ‘scRNA-seq cornea,’ ‘single-cell RNA seq ophthalmology,’ ‘single-cell RNA seq anterior segment,’ and ‘single-nuclei RNA seq anterior segment.’ 126 total publications were identified and 43 articles from 2018 to 2022 were assessed. Additional details on the literature search algorithm are included in the supplemental methods. All articles involving scRNA-seq methods, molecular mechanisms, and clinical importance were included in the analysis. ScRNA-seq studies involving the ocular anterior segment published in foreign languages and non-original review articles were excluded. We also manually filtered out spurious hits that pertained to the ocular posterior segment and eyelid.

## Single-cell rna sequencing experimental design

### Ocular tissue dissociation

To detect novel transcript variants and splicing isoform expression with depth and accuracy, it is first essential to consider how ocular tissues are prepared based on tissue type [16]. A primary concern in post-mortem ocular tissue transcriptomic profiling is preservation of RNA integrity. How ocular tissue cells are isolated can alter transcriptional expression due to inadvertent RNA degradation, failure to isolate cells embedded within collagenous matrix, or transcriptional stress response induction during cell dissociation [28]. Tissues taken from tumour, for instance, are bound by extracellular matrix that renders single cell isolation with minimal disruption of RNA integrity challenging. Haematopoietically-derived cells suspended in the peripheral blood or lymphoid tissues, in contrast, are easier to isolate into individual cells. While there are commercial reagents (such as MACS® Tissue Dissocation Kits, Miltenyi Biotec) available to release cells embedded in extracellular matrix-like structures, it is speculated that these reagents may create artefactual changes in mRNA expression levels prior to single cell isolation [16].

**Fig. 1:**
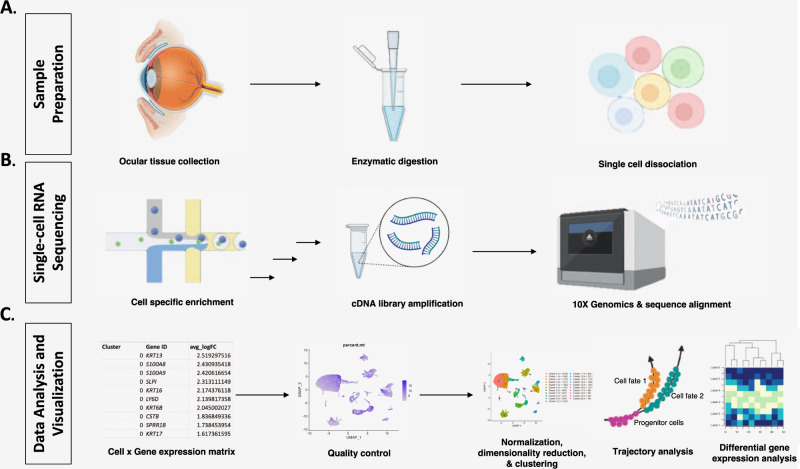
Experimental design for creating an ocular anterior segment single-cell transcriptome atlas. **A** Ocular tissue is surgically excised and transported in saline. Individual cells are enzymatically lysed and dissociated into individual micro-environments. **B** Fluid partitioning is utilized to release mRNA, which is then converted to complementary DNA (cDNA) and amplified using reverse transcription. cDNA libraries undergo sequencing and sequenced reads are aligned to the human genome using various computational tools. **C** The reads undergo quality control and mitochondrial DNA percentage is checked to ensure that only intact cells are included for analysis. An expression matrix indicating the number of reads per gene per cell is obtained. Cells that are transcriptionally similar are grouped into clusters after the data are normalized and undergo dimensionality reduction. Trajectory analysis is used to determine cell fate over time. Cell differential gene expression can be visualized by heatmaps, dot plots, or violin plots (latter two not illustrated).

The method used to isolate individual cells can influence the number of genes detected, number of mRNA transcripts per gene, and resolution of differential splicing. A schematic representation of scRNA-seq experimental design can be found in Fig. 1. Prior studies involving human donors, animal models, and induced pluripotent stem cell-derived (iPSC) organoid models have demonstrated that single cell dissociation should involve enzymatic reagents, such as collagenase and DNAse solution (STEMCELL Technologies), that pose minimal risk of trauma to the cell types in the tissue [1]. Dissociation should also begin as quickly as possible because mRNA from ocular tissues have been previously demonstrated to degrade more quickly after 5 hours have passed, with non-human primate tissues degrading faster than their human counterparts [29, 30]. Furthermore, gene expression levels have been shown to be altered post-mortem, with longer intervals decreasing gene expression in certain cell types [1].

### Single cell capture

Single cell isolation is arguably the most critical bottleneck for obtaining important transcriptomic information about individual cells [16]. The criteria by which cells are isolated can vary from cell morphology, size, and cell type-specific surface markers, the last of which can enable isolation of rare cell populations. Low-throughput methods for single cell isolation include limiting dilution, laser capture microdissection, and micromanipulation, all of which exhibit major limitations for the capture of rare cell types [31]. Micromanipulation is ideal when there are a limited number of cells available or when cells are too fragile that they cannot be left viably intact with other isolation methods, such as fluorescence activated cell sorting (FACS) [32]. While micromanipulation enables direct observation of each cell, it is time-consuming and highly labour-intensive [33]. FACS, in contrast, boasts a remarkably higher throughput. However, FACS, too, comes with limitations, such as the requirement of starting with a large sample volume and the need for utilizing monoclonal antibodies to bind to target proteins of interest. The latter limits the ability to detect novel or rare cell types.

ScRNA-seq studies of the ocular anterior segment ubiquitously utilize high-throughput technologies that integrate single cell isolation and downstream processing to ensure unbiased characterization of cell types at single-cell resolution. Microfluidics devices like Fluidigm’s C1^TM^ Single-Cell Auto Prep System utilize integrated fluidic circuits to enable automated capture of single cells which then undergo cell lysis, RNA reverse transcription to cDNA, followed by polymerase chain reaction (PCR) amplification. A number of studies have employed droplet-microfluidics platforms such as 10X Genomics’ Chromium technology because it enables individual partitioning of thousands of cells and minimize reagent waste, but may isolate certain cell types over others [33]. For instance, studies that have utilized microfluidics instruments to identify rare cell types require an additional enrichment step with either FACS or the MACS® MicroBead technology [31].

### Single-cell RNA sequencing

After single cell isolation, individual cells are lysed to release mRNA. To prevent capture of ribosomal RNA (rRNA), barcoded oligo-dT primers are utilized to capture polyadenylated mRNA [32]. Obtaining accurate genetic information from individual cells requires mRNA conversion to cDNA, which then undergoes PCR amplification or in vitro transcription followed by an additional round of reverse transcription [16]. Amplification of each isolated cell’s genomic content must minimize introduction of artifacts or loss of genomic material. Several scRNA-seq protocols require use of adaptor sequences or unique molecular identifiers (UMIs) prior to amplification to enhance mRNA detection on the sequencing platform, to differentiate unique mRNA transcripts from one another, and to preserve information regarding cellular origin. Barcoded cDNA libraries are prepared in preparation for sequencing, which allows individual reads to be assigned to specific cells and compared with specific genes.

ScRNA-seq protocol selection is dependent on a given study’s research question [3]. Studies that aim to characterize highly heterogeneous tissues benefit from protocols that provide full-length RNA transcript data because of the ability to detect transcripts that are expressed at low levels. Sequencing full-length transcripts also enables detection of splicing isoforms and gene expression quantification. Tag-based protocols, in contrast, enable early multiplexing at higher throughput at the cost of coverage. This can potentially be useful if a study includes multiple samples that would require the use of sample-specific barcodes. While different scRNA-seq protocols demonstrate comparable levels of accuracy in determining the relative abundance of mRNA transcripts within a sample, there has been variable sensitivity for genes that harbour low gene expression levels [16]. However, for transcripts that are moderately or highly expressed, all published protocols have demonstrated high efficacy in determining relative transcript abundance per cell. Droplet-microfluidics methods, which have been used repeatedly in ocular anterior segment studies, are ideal for maximizing throughput or for elucidating rare cell types present in highly heterogeneous tissues. The caveat with droplet-based sequencing protocols is that they exhibit decreased sensitivity for transcripts that have lower expression levels in each sample. Extensive comparisons between a variety of scRNA-seq protocols have been discussed at length elsewhere [34–36].

### ScRNA-seq data processing

**Fig. 2:**
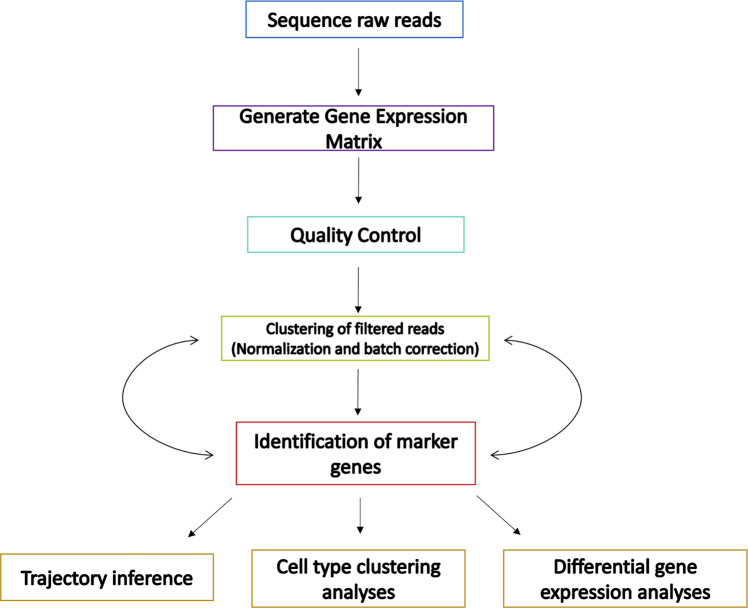
ScRNA-seq bioinformatics pipeline in ophthalmology. Once raw reads are generated, they are aligned and undergo de-duplication to generate an initial gene expression matrix. Quality control (QC) is conducted to remove low-quality reads. The data is also subject to batch correction to estimate confounding factors contributing to technical variability and noise. The raw expression data is normalized to cluster cells by subtype and to minimize cell-specific bias. Dimensionality reduction with principal component analysis is performed to enable cell cluster visualization based on well-established marker gene expression. Cell trajectories can be inferred based on the normalized matrix and by analysing differential gene expression between cell clusters.

Typical scRNA-seq analysis is comprised of several pre-processing steps including read alignment and quantification, quality control, normalization and batch correction, dimensionality reduction, and cell annotation (Fig. 2) [14]. While multiple bioinformatic analysis tools have been developed to perform these initial analysis steps, prominent analysis platforms include Seurat in R [37], Scater in R [38], and Scanpy in python [39], all three of which enable integrated quality control and loading of UMI counts from raw data processing pipelines such as Cell Ranger. Because analysis platforms are often only available in select programming languages, choosing between them is related to one’s proclivity for a particular programming language [39]. The Seurat package in R is a versatile tool that enables integrated analysis of scRNA-seq data with a wealth of scRNA-seq analysis tutorials and community support; however, it has been noted to have limitations in terms of processing speed and efficiency [37]. For analysis of larger and more complex datasets, Scanpy in python is recommended. ‘ScRNA-tools,’ ‘omictools.org,’ ‘awesome-single-cell,’ and the Bioconductor repository are several useful catalogues of tools for scRNA-seq data processing and analysis [16, 40].

The primary aim of pre-processing is to exclude data from cells with poor viability. While there is no consensus on filtering algorithms, most studies to date filter out low-quality cells (indicated by a variable percentage of reads mapping to the mitochondrial genome), rare reads per gene found in a variable number of cells, or presumed doublets. Canonical correlation analysis (CCA) can be used to minimize batch effects [41]. A final gene expression matrix is obtained after cleaning and normalizing the data. Principal component analysis (PCA) using various machine learning algorithms, such as t-distributed stochastic neighbour embedding (t-SNE), is employed to reduce the dimensionality of the data [32]. Dimensionality reduction is essential to cluster cells into subpopulations based on gene expression levels. Clustering also harbours potential pitfalls that can be avoided by applying cluster validation methods discussed at length elsewhere [42].

## Clinical applications

The ocular anterior segment consists of an interconnected set of structures known as the cornea, conjunctiva, iris, ciliary body, crystalline lens, trabecular meshwork (TM), and Schlemm’s canal (SC) [18, 43]. These structures broadly fulfil the essential functions of focusing light onto the retina for visual processing, participating in the accommodation reflex, and modulating immune homoeostasis in the eye [18]. The conjunctiva protects the eye’s surface from environmental insults by producing goblet cell mucins [18]. The cornea, iris, and crystalline lens are responsible for refraction and focusing light onto the retina. Located between the iris and choroid, the ciliary body produces aqueous humour and contains the ciliary muscle, which controls lens shape during accommodation. Aqueous humour exits the anterior chamber through the TM or uveoscleral outflow pathways. Disruption to any of these structures may result in visual impairment from glaucoma, dry eye disease, corneal dystrophies, cataract, uncorrected refractive error, and anterior uveitis, among other pathologies [44].

### Characterization of anterior segment developmental tissue markers

To better understand anterior segment development, researchers have constructed single-cell atlases detailing its developmental tissue markers. Collin et al. analysed 89,897 cells and identified neural crest, mesodermal, and progenitor cells in the developing cornea and conjunctiva in human eyes aged 10-21 post-conception weeks (PCW) [19]. At 10 PCW, significant keratin 13 (*KRT13*) expression in the conjunctival and suprabasal limbal epithelium suggested that the conjunctiva differentiates at an earlier stage compared to corneal epithelium. Corneal samples at 16 PCW exhibited diminished neural crest and mesodermal cell levels and significant Nidogen expression, which suggests that corneal stromal, endothelial, and epithelial basement membrane layers undergo differentiation at this time point. At 20–21 PCW, an increase in fibroblastic corneal endothelial cells was noted, suggesting these cell types serve an integral role in corneal endothelial cell proliferation that may potentially benefit endothelial wound healing. Sun et al. investigated limbal stem cell (LSC) developmental trajectories in vitro at four different time points and found that pluripotent markers POU5F1 (POU class 5 homeobox 1*)*, SOX2 (SRY-Box 2), and NANOG (Nanog homeobox) were most highly expressed at the first time point (Day 0) and steadily decreased by the second time point (Day 7) [45]. TFAP2A (Transcription factor AP-2 alpha), a surface ectodermal marker, and epithelial and limbal stem cell markers were expressed by Day 7. While only 21.82% of LSCs expressed *ABCG2* (ATP-binding cassette super-family G member 2) by day 14, most LSCs (85.67%) expressed this marker by day 21. This data has significantly advanced our understanding of how stem cell populations mediate corneal and conjunctival differentiation.

### Identification of cell types, novel markers, and pathogenic molecular mechanisms in the ocular anterior segment

ScRNA-seq studies feature high accuracy, sensitivity, and throughput, rendering illustrations of the molecular mechanisms in both steady state and disease conditions feasible. The studies listed in Table 2 demonstrate that scRNA-seq is a promising tool for elucidating the pathogenesis of various anterior segment-related diseases and by extension, for developing gene or cell-specific therapies. A comprehensive compilation of publicly available ocular anterior segment scRNA-seq datasets is shown in Table 3.

**Table 2:** ScRNA-Seq studies that include various ocular pathologies of the ocular anterior segment.

Study name	Methodology	Platform	Sample Source	Diagnosis	Number of Cells Sequenced	Year of publication	Molecules/pathways identified	References
Wang et al.	ScRNA-Seq; Bulk RNA Seq	10x Genomics	Human corneal endothelium	FECD	16,942	2022	Nuclear enriched abundant transcript 1 (*NEAT1*) is downregulated in FECD.	[72]
Dou et al.	ScRNA-Seq	10x Genomics	Human adult central cornea	Keratoconus	20,312 (keratoconus corneas), 18,902 (healthy corneas)	2022	*CTSD* and *CTSK* are potential novel markers for keratoconus stromal cells; elevated levels of *YAP1* and *TEAD1* are found in keratoconus stromal cells.	[26]
Wu et al.	ScRNA-Seq	Fluidigm C1 System	Murine sclera	Myopia	93 (49 from form-deprivation sclera, 44 from control eyes)	2018	Hypoxia-inducible factor-1α (HIF-1α) promotes myopia development.	[80]
Alam et al.	ScRNA-Seq; ATAC-Seq; Bulk RNA Seq	10x Genomics	Murine cornea and conjunctiva	Dry eye disease	11,165	2022	γδ T cells are the predominant IL-17 expressing conjunctival immune cell population in Pinkie mouse strain with loss of function RXRα mutation. IL-23 tear concentration is significantly elevated in Pinkie mouse strain compared to wild type strain C57BL/6.	[47]
Ma et al.	ScRNA-Seq	10x Genomics	Human adult conjunctiva	SARS-CoV-2	34,382	2021	*ACE2* and *TMPRSS2* are highly co-expressed in conjunctival goblet cells, thereby facilitating SARS-CoV-2 viral entry and infection.	[54]
Jackson et al.	ScRNA-Seq	10x Genomics	Adult human conjunctival air-liquid interface organotypic model	SARS-CoV-2	8202	2022	Upregulation of NF-κB signalling without activation of antiviral interferon signalling is demonstrated in all conjunctival epithelial cell types infected with SARS-CoV-2, suggesting that genomic expression occurs without productive infection.	[55]
Collin et al.	ScRNA-Seq; scATAC-Seq; ATAC-Seq	10x genomics	Human adult and foetal conjunctiva, limbal, and corneal epithelium	SARS-CoV-2	21,343 (adult), 72,418 (developmental)	2021	Co-expression of *ACE2* and *TMPRSS2* occurs in human adult conjunctival, limbal, and corneal epithelium and is absent in corresponding embryonic and foetal structures up to 21 weeks after conception. TNF, NF-κB, and IFNG regulate the transcriptional profile of *ACE2*^*+*^*/ TMPRSS2*^*+*^ cells.	[53]
Thomson et al.	ScRNA-Seq	10x Genomics	Murine TM and SC	PGC	21,356 (control), 7254 Angpt1ΔNC	2021	PGC in mice is caused by tissue-specific deletion of *Angpt1* or *Svep1* from the TM. Treatment with *Angpt1* recombinant drug promotes development of SC cells, reduces IOP, and RGC loss in a mouse model.	[77]
Collin et al.	ScRNA-Seq; scATAC-Seq	10x Genomics	Human adult central cornea	Keratoconus	2641	2021	Increased collagenase activity in the corneal stroma is a key component of keratoconus pathogenesis; reduced numbers of limbal suprabasal cells and elevated numbers of corneal stromal keratocytes are present in keratoconus	[19]
Gao et al.	ScRNA-Seq; Bulk RNA Seq	GEO Database (Entry number GSE138433)	Human uvea	UM	11,988	2022	142 common mRNA-lncRNA pairs regulated by *MAF*, *RWDD1*, *SLC2A3*, *LDB2*, *CYB2D2*, and *CTNNB1* are dysregulated in UM development.	[84]
Pandiani et al.	ScRNA-Seq	10x Genomics	Human uvea	UM	7890 from non-malignant cells, 7789 from malignant cells	2021	*HES6* is negatively correlated with overall survival and is highly correlated with metastatic risk.	[82]
Hassman et al.	ScRNA-Seq	10x Genomics	Human aqueous humour and blood	Granulomatous uveitis	—	2021	CD4^+^ T cells are the most abundant cell type present in granulomatous uveitis. A 20-fold difference in CD4^+^ T-cell clonality was observed in the affected eye compared to peripheral blood.	[76]
Kasper et al.	ScRNA-Seq	10x Genomics	Human aqueous humour	Acute anterior uveitis associated with HLA-B27 haplotype, infectious endophthalmitis	13,550	2021	HLA-B27 positive uveitis consists of a plasmacytoid and classical dendritic cell infiltrate.	[75]
Bakhoum et al.	ScRNA-seq	Illumina in Drop	Human uvea	UM	17,074	2021	Loss of *BAP1* and *PRC1* is a defining feature of aggressive UM.	[86]

*FECD* Fuchs endothelial corneal dystrophy, *PGC* primary congenital glaucoma, *TM* trabecular meshwork, *SC* Schlemm’s Canal, *IOP* intraocular pressure, *RGC* retinal ganglion cells, *PCW* post-conception week, *UM* uveal melanoma.

**Table 3:** **A**. NCBI Gene Expression Omnibus (GEO) submitted single-cell RNA seq experimental datasets.

Keywords	Dataset description	Species	Number of samples included	NCBI GEO	Sequencing Platform	PMID
Single Cell RNA Seq Cornea	ScRNA seq analysis of corneal limbal cell heterogeneity and interactions with corneal epithelial stem cells in 47,627 cells	*Homo Sapiens*	6 human corneal limbus samples	GSE157474	Illumina NovaSeq 6000	33964410
Single Cell RNA Seq Cornea	ScRNA Seq investigating the development of the human ocular surface from development to adulthood	*Homo Sapiens*	17 developing human cornea and conjunctiva (10-21 PCW); 4 adult cornea/conjunctiva and 8 human cornea-scleral rings	GSE155683	Illumina NovaSeq 6000	33865984
Single Cell RNA Seq Cornea	ScRNA seq investigating corneal epithelial progenitor cells that exhibit colony-forming ability and possess the capacity for self-regeneration in steady and injured states	*Macaca fascicularis*; *Homo sapiens*; *Mus musculus*	2 central corneal epithelial sample from *Mus musculus, Macaca fascicularis*, and *Homo sapiens* under normal conditions, respectively; 2 central corneal epithelial sample from *Mus musculus, Macaca fascicularis*, and *Homo sapiens* obtained 24 hours after surgical injury, respectively	GSE146276	Illumina NovaSeq 6000	*
Single Cell RNA Seq Eye	Construction of the first single cell transcriptome atlas of the adult neotenic axolotl across 19 tissue types and metamorphosed axolotl across 16 tissue types	*Ambystoma mexicanum*	—	GSE201446	HiSeq X Ten; DNBSEQ-T7	35869072
Single Cell RNA Seq Eye	Identification of mechanisms by which human conjunctival cells are infected with SARS-CoV-2 using an organotypic air-liquid-interface model	*Homo sapiens*	3 C16, 2 C15, and 2 C10 human adult conjunctival cell isolates	GSE191232	Illumina NovaSeq 6000	35750043
Single cell RNA sequencing eye	Identification of key cell types found in the human ciliary body	*Homo sapiens*	3 human ciliary body samples	GSE206026	Ilumina NovaSeq 6000	36163311
Single Cell RNA Seq Eye	Identification of various cell types present within the murine ciliary body	*Mus musculus*	2 wild type murine ciliary body samples	GSE178667	Illumina NovaSeq 6000	34717927
Single Cell RNA Seq Eye	Characterization of HLA-B27-associated intraocular leucocytes	*Homo sapiens*	3 HLA-B27 positive, 2 anterior uveitis, and 1 infectious endophthalmitis cell infiltrates	GSE178833	Illumina NextSeq 500	34783307
Single Cell RNA Seq Eye	Identification of splicing patterns in *SF3B1* mutated uveal melanoma that lead to the production of immunogenic neo-antigens	*Homo sapiens*	2 human metastatic UM tumour samples	GSE169609	Illumina NovaSeq 6000	33811047
Single Cell RNA Seq Eye	Identification of transcription factor *HES6* as a driver of UM metastasis and intratumor heterogeneity	*Homo sapiens*	6 human primary UM tumours	GSE138665	Illumina HiSeq 2000; Illumina NextSeq 500	33462406
Single Cell RNA Seq Eye	Characterization of a subset of Th2 cells that express calcitonin gene-related peptide (CGRP) and Il1rl1-encoding ST2 that is implicated in the pathophysiology of allergic conjunctivitis	*Homo sapiens, Mus musculus*	Two CD45 + /CD31- white blood cell fractions taken from murine conjunctiva; One CD45 + /CD4 + white blood cell fractions from the peripheral blood of a human patient; Two CD45 + and CD4 + samples taken from the giant papillae of human patients	GSE203217	Illumina NovaSeq 6000	*
Single-cell RNA sequencing eye	Creation of a transcriptomic atlas of human iPSC-derived lacrimal gland-like organoids	*Homo sapiens*	3 human iPSC-derived lacrimal glands from Day 0, 10, and 20, respectively	GSE174653	Ilumina NovaSeq 6000	35444274
Single-cell RNA sequencing eye	Identification of novel markers of the limbus and corneal stroma identified by analysis in 19,472 cells	*Homo sapiens*	8 healthy donor corneas	GSE186433	Ilumina NovaSeq 6000	34741068
Single-cell RNA sequencing eye	Identification of Schlemm’s canal endothelial cells in Angiopoietin-1 knockout mice	*Mus musculus*	2 wild type murine iridocorneal angle and limbal region samples; 1 neural crest specific Angpt1 knockout murine iridocorneal angle and limbal region sample	GSE168200	Ilumina HiSeq 4000	34663817
Single-cell RNA sequencing eye	Uveal melanoma pathogenesis is driven by loss of Polycomb Repressive Complex (PRC1) identified by analysis of 17,074 cells	*Homo sapiens*	6 human UM tumour samples	GSE160883	Ilumina HiSeq 2500	34518527
Single-Cell RNA Sequencing Eye	Identification of cell types the murine iris using single nuclei RNAseq	*Mus musculus*	3 murine iris control samples, 3 murine constricted iris samples, and 2 murine dilated iris samples	GSE183690	Illumina NovaSeq 6000	34783308
Single nucleus RNA cornea	Characterization of the cell types comprising the cornea, iris, ciliary body, crystalline lens, and aqueous humour outflow pathways	*Homo sapiens*	6 human anterior segment samples	GSE199013	Ilumina HiSeq 2500, Ilumina NovaSeq 6000	35858321

GEO was searched for terms ‘Single Cell RNA Seq Cornea; Cornea Development; ‘Single-cell RNA sequencing eye.’ *Unpublished dataset. **B**. NCBI Gene Expression Omnibus (GEO) submitted single-nucleus RNA sequencing (snRNA-seq) of the ocular anterior segment. GEO was searched for terms ‘Single nucleus RNA Cornea;’ ‘Single-cell RNA sequencing eye.

#### Conjunctiva

Several studies have elucidated cellular heterogeneity and differential gene expression in the conjunctiva. Alam et al. analysed 8909 individual cells from murine conjunctiva obtained from wild-type *C57BL/6* *J (B6)* mice and identified 14 different cell types including macrophages (42.03%), neutrophils (28.76%), monocytes (10.79%), natural killer cells (5.33%), T cells (3.96%), mast cells (1.71%), γδ-T cells (3.19%), and B cells (0.53%), amongst others [46]. Neutrophils exhibited higher expression levels of lipocalin 2 (*LCN2)*, which may play a role in ocular graft vs. host disease (GVHD) and corneal alkali injury. Among monocytes, apolipoprotein E (*APOE)* was the mostly highly expressed gene and plays a role in immune suppression. γδ-T cells highly express interleukin-17 (IL-17), which is purportedly increased in dry eye disease (DED). Type 1 conventional dendritic cells (cDC1) most highly express cystatin 3 (*CST3*), which in Sjögren syndrome is decreased. Alam et al.’s cell-cell interactions analysis found that monocytes and macrophages and classical dendritic cells (cDCs) and myeloid dendritic cells (mDCs) exhibit the strongest ligand-receptor interactions. Regulatory and anti-inflammatory pathways were enriched in monocyte, macrophage, and cDC1 cell populations. Ligocki et al. found that keratin 4 (*KRT4)*, keratin 13 *(KRT13)*, and aquaporin 5 (*AQP5)* are significantly enriched in the conjunctival epithelium, thereby expanding our understanding of conjunctival epithelial markers [22].

##### Dry eye disease (DED) pathogenesis

Alam et al. further expanded on these findings by investigating the molecular mechanisms driving DED pathogenesis in murine disease models [47]. While there are notable differences between mouse and human transcriptional signatures, studies have demonstrated that there is general conservation of the cellular architecture between the two species [44, 48]. Thus, the utility of Alam et al.’s study is contingent upon the extent to which murine conjunctival cell types and gene expression patterns correspond to those found in humans, an area of research that has yet to be further investigated. By comparing 11,165 cells from the C57BL/6 wild type (WT) mouse strain and 7096 cells from the retinoid X receptor α (RXRα) Pinkie mutant strain, Alam et al. found that in the Pinkie conjunctival goblet cells, γδ-T cells expressed IL-17a, IL-17f, IL-23, tumour necrosis factor α (TNF-α), and vascular endothelial growth factor (VEGF). These cytokines are responsible for DED’s cardinal features, including decreased tear volume, conjunctival goblet cell loss, and ulceration. IL-17a was found to be the top differentially expressed gene between WT and Pinkie strains, with the Pinkie strain significantly expressing other IL-17 signature genes such as *LTB (*lymphotoxin-beta)*, CXCR6* (chemokine receptor 6)*, RORC* (RAR-related orphan receptor C), and *IL1RL* (interleukin 1 receptor-like 1). Notably, the authors discovered that a loss of function RXRα mutation is a potential therapeutic target and that RXRα ligand 9-cis retinoic acid (RA) treatment suppresses IL-17 production. Importantly, anti-IL-17 treatment in the murine disease model was also found to mitigate DED progression by reducing matrix metalloproteinase-9 (MMP-9) production and conjunctival goblet cell loss, a finding that has potential implications for developing novel therapeutic strategies in human DED.

##### SARS-CoV-2 infection

Severe acute respiratory syndrome coronavirus clade 2 (SARS-CoV-2) is highly infectious and is transmitted primarily through respiratory droplets and close contact with infected individuals. Viral attachment and entry is facilitated by binding of the virus’ spike (S) protein to cellular receptors found on multiple epithelial cell types in the respiratory airway, such as nasal goblet and ciliated cells [49]. High expression of *ACE2 (*angiotensin converting enzyme 2*)* and *TMPRSS2* (transmembrane protease serine type 2*)* is found in the epithelia of a wide assortment of tissues such as the upper airways, lungs, and oral mucosa [50, 51]. Early on in the SARS-CoV-2 pandemic, there was a paucity of data demonstrating SARS-CoV-2 transmission vis-à-vis the human ocular surface [52]. More recent scRNA-seq studies, however, have provided definitive evidence demonstrating that the conjunctival epithelium plays an integral role in extra-respiratory transmission of SARS-CoV-2.

Collin et al. discovered that human adult conjunctiva is a site for viral infection. Specifically, they found that *ACE2* and *TMPRSS2* co-expression is found only in human adult basal and superficial conjunctival, limbal, and peripheral corneal epithelia and is found at very low levels in their embryonic and foetal counterparts from 7-21 post-conception weeks (PCW) [53]. Importantly, Collin et al.’s findings demonstrate that SARS-CoV-2 infection is facilitated by pro-inflammatory mediators such as TNF, nuclear factor kappa β (NF-Kβ), and interferon gamma (IFNG), suggesting that inflammation is a vital component of SARS-CoV-2 viral entry and propagation in the human ocular surface. Goblet cell gene markers such as *MUC15* (mucin 15*)*, *MUC2 (*mucin 2*), AGR2 (*anterior gradient protein 2 homologue*), VAMP8* (vesicle associated membrane protein 8)*, CLDN7* (claudin 7), *CXCL17* (mucosal chemokine ligand 17), and *SPINT2* (serine peptidase inhibitor, Kunitz type 2) were found to be strongly correlated with *ACE2*. *MUC1* (mucin 1), *MUC4* (mucin 4), *KRT4* (keratin 4), *CEACAM6* (carcinoembryonic antigen cell adhesion molecule 6), and *CXCL17* were found to be highly correlated with *TMPRSS2* and were strongly expressed in superficial and basal conjunctival epithelia. Of note, ingenuity pathway analysis demonstrated that inflammatory responses were dominant pathways enriched in *TMPRSS2*^*+*^ cells. Many of these findings were corroborated by Ma et al., who found that *ACE2* and *TMPRSS2* were most highly co-expressed in conjunctival goblet cells in comparison to other cell types and that *ACE2* correlated genes were strongly associated with immune modulation [54].

Jackson et al. used an ex vivo organotypic air-liquid-interface (ALI) model of conjunctival epithelium to demonstrate that all adult conjunctival cell types are vulnerable to SARS-CoV-2 infection [55]. They found that *ACE2* expression was most elevated in superficial conjunctival epithelium, while *TMPRSS2* was most highly expressed in superficial, basal, and suprabasal conjunctival epithelia. In contrast to Collin et al.’s findings, Jackson et al. found that while conjunctival cells express SARS-CoV-2 RNA after inoculation, there was no significant increase in viral RNA expression over time. Furthermore, they found that conjunctival cells upregulated TNF, interleukin-1 (IL-1), interleukin-6 (IL-6), and NF-Kβ activity and downregulated antiviral IFN signalling, preventing active infection from ensuing after viral entry. Armstrong et al. summarize these findings in their SARS-CoV-2 infection of the ocular surface review [56].

#### Ciliary body

More recently, researchers have set out to identify cell interactions and cell-specific discriminative gene markers in the ciliary body that potentially contribute to the pathogenesis of glaucoma and uveitis. Lou et al. obtained a total of 14,563 individual human ciliary cell single-cell transcriptomes and identified 14 major cell types [57]. They discovered that the majority of ciliary body immune cells are monocytes, macrophages, neutrophils, and mast cells. The ciliary epithelium expresses the retinal and anterior neural fold homeobox (*RAX*) gene, which distinguishes it from other ciliary cells. Of note, only 260 out of 16,088 genes were differentially expressed in PE and NPE transcriptomes, suggesting that these cells display similar gene expression profiles. Lou et al. was also able to identify 8079 cell-cell interactions between fibroblast, PE, NPE, monocyte, and EC cell types, with most interactions revolving around growth factor and hormone release. Interestingly, the study identified interactions between *ICAM1 (*intercellular adhesion molecule 1) and *IL15RA (*interleukin 15 receptor subunit alpha) as common biomarkers in immune-mediated uveitis. They also found that fibroblasts, EC cells, and SMC cells strongly expressed *ANGPT1 (*angiopoietin 1) and *TEK (*TEK receptor tyrosine kinase), an interaction that is strongly implicated in primary congenital glaucoma pathogenesis. Youkilis and Bassnett’s study reiterated these findings by analysing 10,024 murine ciliary epithelial single cells [58]. Although many cell clusters corresponded to the adjacent neural retina, two clusters corresponding to NPE and PE were identified by their canonical markers *BEST2 (*bestrophin 2*)* and *SLC4A4 (*solute carrier family 4 member 4*)*, respectively. Corroborating Lou et al.’s findings, Youkilis and Bassnett found that the PE mostly expressed melanin synthesis genes and the NPE expressed collagen synthesis genes, supporting its ECM maintenance role.

Additionally, Gautam et al. found that *COL9A2 (*collagen type IX alpha 2 chain*)-*high ciliary body cells (CBCs) and pigmented CBCs were transcriptionally similar to putative stem cell populations due to high expression levels of eye field transcription factors, *PAX6* (paired box protein Pax-6) and *SIX3* (six homeobox 3) [18]. This suggested that both cell types may originate from putative stem cells, a phenomenon that appeared to be conserved across human and porcine species. *COL91A* CBCs were found to highly express *CPAMD8* (C3 and PZP-like alpha-2 macroglobulin domain containing 8), a gene co-expressed in porcine iris cells and associated with periocular mesenchymal development.

#### Lens

To date, there is a paucity of lens scRNA seq data. Single-cell knowledge of lens development and gene regulatory mechanisms has been mostly derived from vertebrate model systems, such as from zebrafish. Farnsworth, Posner, and Miller analysed 44,102 cells taken from zebrafish embryos 1,2, and 5 days after fertilization and identified β-crystallin *CRYBA1A*, lactose-binding *GRIFIN* (encodes galectin-related inter-fibre protein), and the aquaporin *MIPA* (major intrinsic protein of lens fibre) as lens fibre cell marker genes [59]. They additionally identified a transcription factor, *FOXE3 (*forkhead box E3*)*, as a lens epithelial cell marker and found alterations in epithelial gene expression between the first and second day after fertilization, which correlates with transition from lens to simple cuboidal epithelia and secondary fibre cell formation. Crystallin alpha A (*CRYAA)* and γM-crystallin expression was exclusively identified in lens fibre cells and αB-crystallin genes were expressed in non-lens tissues during development. Notably, Farnsworth, Posner, and Miller use scRNA-seq data to confirm that two genes, *CELF1* (CUGBP Elav-like family member 1*)* and *RBM24A* (RNA binding motif protein 24a) are strongly expressed in the lens and can be potential targets for CRISPR-Cas9 editing and morpholino-induced translation blocking, both of which have caused developmental defects in the eye [60, 61].

#### Iris

ScRNA-seq studies of iris structure, function, and pathology are of paramount importance because the iris is a potential cell source for ocular auto-transplantation and engineering [21]. Clinically, the iris is an inflammatory site in anterior uveitis and is one of the primary tissues affected in coloboma or aniridia. Gautam et al. found that the iris is a heterogenous assortment of cells derived from different developmental origins [18]. Along with the ciliary body, the most dominant cell type found in the iris is fibroblasts, which were further sub-categorized into maternally expressed 3 (*MEG3*)-high fibroblasts, matrix glia protein (*MGP*)-high fibroblasts, Wnt inhibitory factor (*WIF1*)-high fibroblasts, ribosomal genes high fibroblasts, and fibroblasts. MEG-3 expressing fibroblasts were identified as a notable cell type involved in glaucoma pathogenesis. Gautam et al. identified *MDK* (midkine) as a gene marker for putative iris stem cell populations. They found that significant numbers of transcription factors (TFs) *PAX3* (paired box 3), *MITF* (melanocyte inducing transcription factor), and *SOX10* (SRY-box transcription factor 10) were located in iris melanocytes, suggesting that they play an important role in fibroblast to melanocyte trans-differentiation.

#### Cornea

##### Corneal Limbus

Early cornea scRNA-seq studies focused primarily on characterizing rare stem cell populations in the corneal limbus, the transition zone between the conjunctiva and cornea that functions to regulate corneal epithelium homoeostasis, particularly during wound healing [62]. Limbal stem cell (LSC) characterization has been challenging due to their relative scarcity and bulk RNA sequencing’s technical limitations [22]. Elucidating the different cell types in the heterogenous limbal niche could be extremely valuable for autologous limbal tissue, limbal allograft, conjunctival, and tissue engineered corneal epithelial transplantation [63]. Regeneration of a healthy corneal epithelium is contingent upon successful corneal limbal and limbal stem and progenitor cell transplantation.

Human embryonic stem cells (hESCs) have been employed to create tissue engineered corneal epithelium, a method that has promising implications for treating limbal stem cell deficiency (LSCD) cause by chemical or thermal injury, Stevens-Johnson syndrome, radiation injury, microbial infection, aniridia, or acute ocular trauma [63]. He et al. profiled 29,812 individual cells at four different time points (stage I-IV) during the entire differentiation process using trajectory inferencing. Embryonic stem cell markers *OCT4* (octamer-binding transcription factor 4) and *NANOG* (NK2-family homeobox transcription factor) were found to be highly expressed in stage I whereas *PAX6* (paired box protein Pax-6) was highly expressed from stage II to IV and *TP63* (tumour protein P63) was highly expressed in stage III. The Wnt, β-catenin, Notch, and TGF-β signalling pathways were all found to be upregulated until Stage IV. Importantly, He et al. was able to demonstrate that hESC-derived epithelial cells can be transplanted onto a LSCD rabbit model with minimal corneal opacification and neovascularization.

In a different study, Sun et al. utilized human pluripotent stem cell-derived limbal stem cells (hPSC-derived LSCs) to identify tetraspanin 7 (*TSPAN7)* and SRY-box 17 (*SOX17)* as novel LSC markers [45]. Pseudotime analysis demonstrated that limbal progenitor cells (LPCs) differentiate into five other differentiated limbal and corneal epithelial cell types. Li et al. corroborated these findings with 16,360 healthy human limbal basal epithelial cells [25]. Using scRNA-seq and trajectory analysis, they were able to trace LSC developmental trajectory to LPCs and transient amplifying cells (TACs), which then differentiate into post-mitotic cells (PMCs) and terminally differentiated cells (TDCs). Notably, Li et al. functionally validated *TSPAN7* and *SOX17* as LSC marker genes using human limbal epithelial cells (HLECs) in vitro culture models, immunofluorescent staining, and RNA interference by silent RNA (siRNA), finding that these genes become significantly upregulated during wound healing. Single-cell regulatory network inference and clustering (SCENIC) analysis revealed that there are 51 strongly expressed transcription factors regulating LSC differentiation and regeneration, including those that are involved in the Wnt/β-catenin, PI3K/Akt, Notch, and Hippo signalling pathways. Dou et al. added to these findings by identifying two undifferentiated cell states characterized by high *TP63* and *CCL20* (cysteine-cysteine motif chemokine ligand 20) expression and two differentiated cell states characterized by *GPHA2* (glycoprotein hormone subunit alpha 2) and *KRT6B* (keratin 6B) expression [64].

Using a murine model, Altshuler et al. identified novel gene markers associated with outer limbal basal epithelial cells and inner limbal basal epithelial cells [24]. The former was found to be associated with keratin 15 (*KRT15)*, *IFITM3* (interferon induced transmembrane protein 3), and *CD63* whereas the latter highly expressed *KRT15-GFP*, *ATF3* (activating transcription factor 3), and *MT1-2* (melatonin receptors 1–2). Limbal suprabasal cells were found to highly express *PRDM1* (PR domain zinc finger protein 1). The findings from their study suggest that inner limbus cells are significantly involved in homoeostasis maintenance while outer limbal cells are involved in maintaining a reservoir of quiescent limbal stem cells (qLSCs), which are regulated by T cells during wound healing. Knockdown experiments using endoribonuclease-prepared silencing RNA (esiRNA) further provided evidence that *GPHA2* and *IFTM3* (encodes interferon induced transmembrane protein 3) are involved in maintaining the LSC undifferentiated state.

Kaplan et al. discovered novel regulatory mechanisms by which corneal epithelial cells maintain quiescence [20]. In blecin-1-deficient mice, they demonstrated that decreased *PDZ-binding kinase* (*PBK*) levels contribute to dysregulated corneal epithelial cell G2/M cell cycle arrest, thereby resulting in compromised wound healing [65]. *LRIG1* (leucine-rich repeats and immunoglobulin-like domains protein 1) was identified in stem/early TACs while *MKI67* (marker of proliferation Ki-67) was found to be highly expressed in TACs. *TXNIP* (thioredoxin-interacting protein) was identified as a novel stem and early TAC marker. *TXNIP* uses *p27*^*kip1*^ (cyclin-dependent kinase inhibitor 1B) to promote G0/G1 cell cycle arrest. These findings are valuable for identifying corneal wound healing and regeneration therapeutic targets.

Català et al.’s healthy human corneal single-cell atlas provides unprecedented detail of novel gene markers that enable identification of LSCs, TACs, and corneal stromal cells, information that can be utilized to advance cell replacement and corneal regenerative therapies [66]. They demonstrated that both basal corneal limbal epithelial cells and TACs highly express *CAV1* (caveolin 1), *CPVL* (carboxypeptidase vitellogenic-like), and *HOMER3* (homer scaffold protein 3), suggesting that these may be potential LSC markers. *CXCL14* (chemokine ligand 14) was identified as a suprabasal and superficial limbal cell marker, whereas *CKS2* (cyclin-dependent kinases regulatory subunit 2), stathmin *(STMN1)*, and *UBE2C* (ubiquitin conjugating enzyme E2 C) were found to be TAC markers. Distinct from adult stem cells, TACs have the capacity to produce many functionally mature cells during both development and regeneration. To obtain a more comprehensive understanding of TAC transcriptomic heterogeneity, Li et al. analysed 16,360 limbal basal cells and identified exclusive expression of proliferation-related and cell-cycle dependent genes such as *RRM2* (ribonucleotide reductase regulatory subunit M2), *TK1* (thymidine kinase 1), *CENPF* (centromere protein F), *NUSAP1* (nucleolar and spindle-associated protein 1), *UBE2C*, and *CDC20* (cell division cycle protein 20) [67]. To add to this, Ligocki et al. found *GPHA2*, *SCRG1* (stimulator of chondrogenesis 1), *FRZB* (frizzled-related protein), *LECT1* (chondromodulin-1), *NPPC* (natriuretic peptide precursor C), and cadherin 19 (*CDH19)* to be significantly upregulated in early LPCs [22]. *MMP10* (matrix metallopeptidase 10) was found to be a gene marker highly expressed in both early and late LPCs.

Finally, Li et al. performed scRNA-seq on adult human corneal limbus cells to investigate seven immune cell types residing within the cornea that are necessary for maintaining homoeostasis and ensuring protection from environmental insults and infections [68]. These cell populations and their relative percentages included naïve T cells (17.1%), monocytes (12.8%), double-negative T cells (17%), CD8 + T cells (19.1%), dendritic cells (DCs) (5.1%), macrophages (16.5%), and basophils (12.2%) based on canonical gene markers. The DC population mostly consisted of mature regulatory dendritic cells (mregDCs), which were identified by *LAMP3* (lysosomal associated membrane protein 3) and *BIRC3* (baculoviral IAP repeat containing 3) upregulation as well as pro-inflammatory cytokine IL1B, IL-15, and IL-23A expression. The most highly expressed genes were involved in mediating inflammatory responses and cellular development.

##### Adult human cornea

Unfortunately, corneal cell replacement therapies using LSC transplantation do not extend to corneal stromal or endothelial regeneration, which are prerequisites for whole corneal regeneration. To better understand *all* corneal cell types found in the developing and adult human cornea, several researchers have set out to create healthy adult human corneal single cell atlases. Català et al. sequenced 19,472 corneal cells and identified *NNMT* (nicotinamide N-methyltransferase) to be a novel corneal stromal keratocyte marker [66]. Corneal endothelial cells in cluster En0 were found to highly express *COL4A3* (collagen type IV alpha 3 chain), which suggests that these particular cells play an essential role in Descemet’s membrane homoeostasis.

Ligocki et al. analysed 16,234 cells and identified 16 clusters comprised of conjunctival epithelial cells (20.1%), stromal cells (15.2%), Langerhans cells (0.8%), superficial mature epithelial cells (37.5%), basal epithelial cells (12.2%), transitional epithelial cells (6.8%), melanocytes (0.3%), vascular endothelial cells (0.2%), corneal endothelial cells (0.2%), early and late limbal progenitor cells (1.6% and 3.3%, respectively), and TACs (1.7%) [22]. In corneal endothelial cells, *CA3* (carbonic anhydrase 3) and *SCL4A11* (solute carrier family 4 member 11) were validated to be important gene markers and Wnt and fibroblast growth factor (FGF) pathways were found to be enriched, the latter of which were noted to play an integral role in in vitro corneal endothelial cell differentiation and proliferation of grafts. The corneal stroma, on the other hand, was found to be composed of classic keratocyte markers, keratocan (*KERA)*, lumican (*LUM)*, and decorin (*DCN)*, all of which are associated with stromal corneal dystrophies. Stratifin (*SFN)*, keratin 5 (*KRT5), TACSTD2* (tumour-associated calcium signal transducer 2), and *S100A14* (S100 calcium binding protein A14) were found to be corneal epithelial cell gene markers. Langerhans cells were found to be enriched for *MSR1* (macrophage scavenger receptor 1), *VSIG4 (*v-set and immunoglobulin-domain containing 4*)*, and *PTPRC* (protein tyrosine phosphatase receptor type c) whereas melanocytes were found to be characterized by the canonical markers *TYRP1* (tyrosinase-related protein 1), *DCT* (dopachrome tautomerase), and *PMEL* (promelanosome protein). Claudin 5 (*CLDN5), PECAM1* (platelet and endothelial cell adhesion molecule 1), and *COL4A2* (collagen type IV alpha 2 chain) were found to be highly expressed in vascular endothelial cells. A wide assortment of corneal dystrophies were found to be associated with *TGFBI* (transforming growth factor beta induced) mutations.

Gautam et al. identified 7 different cell clusters in the human adult cornea [18]. By creating a disease map across various ocular tissues, they also demonstrated that gelsolin (*GSN)* mutations are significantly associated with corneal dystrophies. *TGFBI* epithelial cells express annexin A1 *(ANXA1)* in inflammatory states and *ELF3* (ETS transcription factor 3) is expressed during differentiation. Consistent with other findings, they demonstrated that *ACE2* and *TMPRSS2*, along with other cell surface proteins that serve as entry points for various viruses such as SARS-CoV-2, are expressed in corneal conjunctival cells. Eriksen et al. used an organoid model derived from hESCs called, “self-formed ectodermal autonomous multizone (SEAM)” to demonstrate that SARS-CoV-2 has a proclivity for infecting the adult corneal limbus, in addition to infecting the cornea, sclera, and RPE [69].

Additionally, Collin et al. analysed 21,343 transcriptomes and identified gene markers associated with stromal, epithelial, immune, endothelial, red blood cell, and melanocyte populations in the adult human cornea [19]. Their pseudotime analysis corroborated Sun et al.’s findings. Like Altshuler et al., Collin et al. identified that loss of expression of *GPHA2* was found to induce LSC differentiation, a finding that was further corroborated with RNA interference and in vivo corneal limbal dysplasia studies. Limbal progenitor cells and immune cells were observed to exhibit significant cell-cell interactions, with the latter releasing TNFα and IL1β to induce differentiated corneal epithelial cell apoptosis. These findings support the idea that pro-inflammatory mediators can regulate limbal progenitor cell proliferation.

Finally, scRNA-seq has been utilized to discern different corneal myeloid cells [70]. Wieghofer et al. found that corneal and ciliary body macrophages were transcriptionally distinct from retinal macrophages. By employing an embryonic fate mapping model, corneal resident macrophages were found to arise from yolk sac or foetal liver embryonic precursors in a murine model. Corneal macrophages demonstrated a high turnover rate in comparison to ciliary body macrophages, suggesting that there is ongoing replenishment of corneal macrophages (possibly from circulating blood cells) that continues throughout adulthood.

##### Corneal nerve repair

Another scRNA-seq clinical application has been to identify important corneal nerve repair mechanisms. As one of the most innerved human tissues, the cornea plays host to non-myelinated Schwann cells (nm-SCs) whose cell-cell interactions and signalling pathways are unclear [71]. By analysing 6546 single cells from rabbit central cornea, Bargnana-Mohan et al. identified strong *SCN7A* (sodium voltage-gated cannel alpha subunit 7), *MATN2* (matrilin 2), *NGFR* (nerve growth factor receptor), *NCAM1* (neural cell adhesion molecule 1), and *PAX3* (paired box gene 3) expression in nm-SCs. Cross-species validation with adult murine cornea revealed significant nm-cSC *NCAM1* expression. Notably, novel Dickkopf-related protein 1 (*DKK1*) and proteolipid protein 1 (*PLP1*) expression in nm-cSCs were also identified, further expanding our understanding of nm-cSC protein biomarkers and their role in corneal nerve injury repair.

##### Keratoconus pathogenesis

ScRNA-seq has been used to investigative disease mechanisms in keratoconus, a progressive condition that causes the cornea to adopt a conical shape, resulting in progressive visual impairment and possibly vision loss. Collin et al. sequenced 2641 cells from two keratoconus patients and concluded that collagenase activation, decrease in limbal suprabasal cells, and increase in corneal stromal keratocytes are key disease pathogenic mechanisms [19]. Collagen synthesis, Wnt signalling pathway, serine protease inhibitor, mitochondrial, and TGFβ genes were found to be significantly downregulated whereas genes involved in ECM degradation, apoptosis, and epithelial to mesenchymal transition were upregulated. EIF2 and mTOR signalling, oxidative phosphorylation, and mitochondrial dysfunction were the most significant molecular pathways identified in keratoconus pathogenesis by scRNA-seq analyses. These findings were previously reported to be essential to keratoconus progression in proteomic studies.

Dou et al. analysed gene expression patterns of 20,312 cells from keratoconus patient central corneas and similarly found that 340 genes were upregulated and 422 genes were downregulated [26]. Importantly, the authors identified cathepsin D (*CTSD*), cathepsin K (*CTSK*), YES-associated protein 1 (*YAP1*), and TEA domain transcription factor (*TEAD1*) as novel biomarkers in keratoconus stromal cells. Upregulated genes were primarily involved in collagen metabolism, ECM disassembly, cornification, keratinization, and keratinocyte differentiation whereas the downregulated genes were primarily associated with mRNA metabolic processes and stress response. These findings provide a potential explanation for corneal stromal thinning and ECM dysregulation during keratoconus progression. SCENIC analysis revealed that five transcription factors (*NFKB1*, *EGR1*, *BCLAF1*, *CEBPD*, and *XBP1*) and their target genes (*FN1*, *COL12A1*, *TIMP3*, and *FBLN5*) were decreased in keratoconus corneal stromal cells. Importantly, Dou et al. discovered that keratoconus can be potentially classified as an inflammatory condition, given the significant pro-inflammatory interleukin and chemokine upregulation such as IL23A and CXCL1 in keratoconus corneas.

##### Fuchs endothelial corneal dystrophy (FECD) Pathogenesis

ScRNA-seq has helped resolve the molecular landscape of FECD and identify potential therapeutic targets. As a corneal endothelial dystrophy, FECD is an age-related disease caused by degenerating corneal endothelial cells that may cause corneal oedema [72]. While the exact aetiology is unknown, its pathogenesis is hypothesized to involve a combination of genetic and environmental factors. Currently, corneal transplantation is the only viable FECD treatment available, but allograft rejection, cornea donor tissue shortages, and cornea graft failure have prompted researchers to investigate other alternative therapeutic strategies. Wang et al. found that loss of expression of a long non-coding RNA (lncRNA) known as nuclear enriched abundant transcript 1 (*NEAT1)* results in human corneal endothelial cell loss, which is essential to FECD pathogenesis [72]. A UVA-induced mouse FECD model was used to validate *NEAT1* knockdown as integral to FECD pathogenesis. Interestingly, the authors employed a CRISPR-activated adenoviral delivery system to overexpress *NEAT1* in vivo in murine corneal endothelial cells and discovered that this could preserve the cells’ morphology, architecture, and density, thereby preventing FECD development despite UVA irradiation exposure.

#### Aqueous humour

Additionally, recent studies have attempted to create aqueous humour single-cell transcriptomic profiles in patients with uveitis, a condition characterized by ocular inflammation associated with myriad infectious, systemic, or idiopathic aetiologies [73, 74]. Anterior uveitis is the most common form of uveitis, comprising more than 50% of uveitis cases [75]. It primarily affects the iris and ciliary body and is considered a risk factor for cataract, glaucoma, and macular oedema development. To elucidate HLA-B27-associated acute anterior uveitis pathogenesis, Kasper et al. captured 13,550 cells from fresh aqueous humour fine needle aspirates obtained from 6 patients [76]. They found that the aqueous humour contains 13 cell types of lymphoid (60%) and myeloid (40%) origins. Plasmacytoid and classical dendritic cells (cDCs) were found in higher concentrations in HLA-B27-associated uveitis aqueous humour samples. Cell-cell interaction analysis demonstrated that cDCs were strongly active in HLA-B27-associated acute anterior uveitis, with cell surface receptors *CD74* and *HLA-E* interacting with surface receptors such as *MIF* and *KLRC1*, all of which play critical roles in antigen presentation and immune modulation. Of note, a patient with infectious endophthalmitis was found to have aqueous humour comprised of cells exclusively from the myeloid lineage. High levels of IL-6 and IL-1 receptor antagonist (IL-1RA) were found in uveitis and endophthalmitis patients, but cytokine levels were found to exhibit high inter-patient variability.

Hassman et al. similarly utilized scRNA-seq to identify immune cell populations in active granulomatous uveitis, a type of uveitis associated with corneal endothelial ‘mutton-fat keratic’ deposits and nodules embedded in the iris and TM [76]. They discovered that CD4^+^ T cells were the most abundant immune cell type found in the granulomatous uveitis aqueous humour, with significant cell type interpatient variation. In comparison to peripheral blood myeloid cells, intraocular myeloid cells expressed higher major histocompatibility complex II levels. Intraocular natural killer (NK) cells expressed lower *FCGR3A* (Fc gamma receptor IIIa)*, GZMB* (granzyme B), and *CXCR3* (chemokine receptor 3) levels compared to peripheral blood NK cells, indicating that these cells are less cytotoxic than their peripheral blood counterparts.

#### Trabecular meshwork and Schlemm’s canal

The iridocorneal angle is comprised of the trabecular meshwork (TM) and Schlemm’s canal (SC). Defects in resident cell types in these tissues can contribute to decreased aqueous humour drainage and subsequent intraocular pressure (IOP) elevation, which can potentially lead to ocular hypertension and glaucoma pathogenesis. The cellular composition of these tissues has been challenging to study because of their highly complex architecture and small size [44]. In the past two years, scRNA-seq has been employed to elucidate the TM and SC’s cellular composition and the signalling pathways that direct their physiology and function at a molecular level. These studies have provided unprecedented insight into glaucoma-relevant genes and potential therapeutic targets for gene therapy targeting both congenital and acquired forms of glaucoma.

Patel et al. captured 8758 cells from eight human donors and identified 12 cell types and 17,757 genes [23]. They found that the TM was primarily comprised of Schwann cell-like cells (28.81%), smooth muscle cells (13.56%), TM2 myofibroblast-like cells (12.50%), TM1 fibroblast-like cells (24.56%), melanocytes (6.76%), macrophages (4.12%), pericytes (2.72%), vascular endothelial cells (2.37%), T/NK cells (1.67%), lymphatic-like endothelial cells (1.42%), myelinating Schwann cells (1.16%), and epithelial cells (0.34%). In contrast, the cell types comprising the SC consisted of both lymphatic and vascular endothelial-like cell phenotypes that most highly expressed *FLT4* (fms related receptor tyrosine kinase 4), fibronectin (*FN1)*, and *FLT1*. Notably, the researchers found that several gene markers associated with elevated intraocular pressure were highly expressed in the TM and SC. TM cell types highly expressed myocilin (*MYOC)* and *ANGPT1*, whereas lymphatic and vascular endothelial cell clusters that were primarily found in the SC highly expressed *CAV1* and *CAV2*. Schwann cell-like and melanocyte cell clusters highly express *ENPP2* (ectonucleotide pyrophosphatase/phosphodiesterase 2), providing confirmatory evidence of *ENPP2* (encodes autotaxin) inhibitors’ efficacy in lowering mice and rabbit IOP. Importantly, this study’s findings could be used to validate the molecular mechanism of rho-kinase inhibitors for decreasing outflow resistance in glaucoma.

Thomson et al. utilized scRNA-seq to demonstrate that loss-of-function mutations in angiopoietin (*ANGPT1)-TEK* (tunica interna endothelial cell kinase) or *SVEP1* (sushi, von Willebrand factor type A, EGF, and pentraxin domain containing 1) is crucial to primary congenital glaucoma (PGC) development in murine models [77]. Their study demonstrates that TM cells contribute to SC development, suggesting that “cross-talk” between the TM and SC regulates IOP homoeostasis. Importantly, Thomson et al. identified twenty-one genes in SC endothelial cells and thirty genes in TM cells as glaucoma gene markers. Their study’s clinical utility lies in the identification of *ANGPT1-TEK* signalling as a potential glaucoma therapy target and demonstrating that a recombinant *ANGPT1*-mimetic fusion protein can reduces IOP elevation and retinal ganglion cell (RGC) loss in a murine PGC model.

Van Zyl et al. expanded on these findings by demonstrating conservation of select cell types and gene markers across human, cynomolgus macaque (*Macaca fascicularis*), rhesus macaque (*Macaca mulatta*), pig (*Sus scrofa*), and mouse (*Mus musculus*) species, suggesting that there is variable efficacy in developing therapeutic targets in human patients using non-human vertebrate models [44]. They found that *MYOC* (myocilin), *FOXC1* (forkhead box C1)*, PITX2* (paired like homeodomain 2), and *CYP1B1* (cytochrome P450 family 1 subfamily B) are both strongly implicated in juvenile glaucoma and ocular hypertension and highly expressed in TM and ciliary muscle cell types. Notably, *OPTN* (optineurin), *ATXN2* (ataxin-2), *TMCO1* (transmembrane and coiled-coil domain-containing protein 1), and *SIX6* (homeobox 6) were strongly expressed in RGCs as opposed to cells of the aqueous humour outflow pathways. *CAV1, CAV2*, and *POU6F2* (POU class 6 homeobox 2) were highly co-expressed in the anterior segment and retina, suggesting that there are secondary pathways that are non-IOP related that can lead to glaucoma development.

The primary caveat with these studies’ findings is that they include a portion of ciliary muscle and scleral spur, which resulted in a relatively small SC endothelial cell yield [78]. For instance, van Zyl et al.’s group recovered only 25 SC endothelial cells out of 13,833 sequenced cells. To address the limited SC endothelial cell capture, Thomson and Quaggin recently published an optimized protocol to enhance corneal removal and SC endothelial cell capture during dissection. Future studies could potentially utilize this protocol to update SC scRNA-seq analyses.

#### Sclera

While the sclera does not explicitly form the ocular anterior segment, it attaches to the corneal limbus and transitions anteriorly to form the cornea [79]. LSCs are located in close proximity to the sclera [45]. Gautam et al. demonstrated that the most populous cell type present in the human sclera are fibroblasts with significant overlap in cellular composition with the choroidal layer [18]. The sclera is the site of ECM remodelling in patients with uncorrected myopia, a condition that is a significant risk factor for glaucoma and cataract development. Wu et al. employed scRNA-seq of 93 scleral cells to identify hypoxia-inducible factor-1α (HIF-1α)-, eIF2-, mTOR- signalling pathway upregulation as a key mechanism for myopia progression in murine models vis-à-vis fibroblast to myofibroblast trans-differentiation [80]. They found that *SMAD4* (SMAD family member *4*) and *Hif1a* (hypoxia-inducible factor 1-alpha) are transcription factors involved in modulating the TGF-β and HIF-1α signalling pathways, respectively. By identifying signalling pathways essential to myopia development, Wu et al. found that anti-hypoxic drug salidroside and formononetin application can downregulate HIF-1α expression and eIF2α/mTOR phosphorylation, thereby halting myopia progression in humans.

#### Uvea

ScRNA-seq’s value in ophthalmology is concretely demonstrated through its applications for elucidating the molecular mechanisms of uveal melanoma, a rare and potentially lethal ocular tumour that harbours a strong metastasis predilection [81]. In contrast to bulk RNA-sequencing, scRNA-seq provides unprecedented information regarding UM’s cellular composition, tumour microenvironment, and rare tumour cell populations associated with poorer prognosis [82]. ScRNA-seq has been used to investigate alterations in gene expression patterns that give rise to UM intratumor heterogeneity (ITH), information that can further enhance our understanding of drug resistance and relapse and assist in targeted immunotherapy or siRNA therapy development. Strub et al. summarizes these data in their review on the clinical applications of scRNA-seq in UM [83].

Gao et al. analysed sequencing data obtained from 11,988 tumour cells and was able to identify 11 different cell clusters in UM, 5 of which are associated with poorer prognosis [84]. Durante et al. analysed 59,915 single cells from eight primary and three metastatic UM tumours and identified *LAG3* (lymphocyte-activation gene 3) as a potential target for immune checkpoint blockade in metastatic UM [85]. They were also able to quantify the density of infiltrating immune cells present in each UM molecular subtype, which depends on the tumour’s gene expression profile, chromosome copy-number variations, and mutations. Metastatic UM was found to harbour significant immune cell infiltration comprised of M2-type macrophages and few NK cells. UM tumours with chromosome 3 monosomy exhibited higher M2-type macrophage concentration, supporting prior evidence that associated chromosome 3 loss with poorer prognosis. These findings can be particularly useful for targeted immunotherapy development. Pandiani et al. analysed 7890 single cells and identified *HES6* (Hes family bHLH transcription factor 6) as a novel gene marker that is correlated with chromosome 3 loss, both of which are strongly associated with UM patient metastatic risk and poorer prognosis [82]. They demonstrated that *HES6* inhibition by siRNA can prevent primary uveal melanoma cells to form a colony, which suggests that *HES6* may be a validated siRNA therapy target. Bakhoum et al. utilized scRNA-seq to demonstrate that PRC1 (polycomb repressive complex 1) loss results in chromosomal instability and is partly responsible for driving aggressive UM progression [86].

ScRNA-seq has also provided unprecedented insight into splicing mechanisms driving UM pathogenesis. Bigot et al. identified splicing factor gene *SF3B1* (splicing factor 3B subunit 1) mutation as a mechanism for tumour neoantigen production that is aberrantly expressed in tumours specific to each individual patient [87]. These neoepitopes were shown to be detected by the patients’ CD8^+^ T cells. These findings help elucidate how metastatic UM tumours resist anti-checkpoint inhibitor therapy. They also lend credence to novel methodologies such as “immunopeptodomics” to identify tumour neoantigens and contribute to vaccine development that targets *SF3B1*^*mut*^-related epitopes or adoptive cell transfer therapy transducing T cells with specific T cell receptors (TCRs) towards tumour neoepitopes.

## Single-nuclei rna sequencing (snrna-seq) of the ocular anterior segment

An alternative approach to scRNA-seq is single-nuclei RNA sequencing (snRNA-seq), which is performed on single nuclei rather than intact cells. While nuclei contain significantly reduced mRNA levels compared to whole cells, the number of genes detected per cell are comparable between snRNA-seq and scRNA-seq if mapped intronic reads are included in the analysis [88]. SnRNA-seq can be used to enhance detection of rare cells and cells embedded in collagenous matrix, making snRNA-seq a suitable methodology for studying tumour cell populations. Single nuclei isolation employs the use of tissue homogenization, which can inhibit RNA degradation during the nuclei purification step. In contrast to scRNA-seq, which often involves enzymatic dissociation of single cells, snRNA-seq is thought to minimize computational bias associated with dissociation-induced artefactual transcriptional changes. Tissue flash-freezing during nuclear dissociation can also prevent new gene transcription. While Wu et al. has shown that single-cell and single-nucleus methodologies exhibit comparable sensitivity levels, Liang et al. has illustrated that snRNA-seq datasets show enhanced power for demonstrating disease-gene associations compared to scRNA-seq analyses [28, 88]. However, a notable limitation of snRNA-seq is that it cannot be utilized to capture information on the transcriptional landscape of cytoplasmic RNA [21].

To date, there are two snRNA-seq studies pertaining to the ocular anterior segment. Using snRNA-seq data obtained from 34,357 murine iris nuclei, Wang et al. identified two iris stromal cell types and two iris sphincter cell types in healthy iris tissue [21]. They elucidated transcriptomic signatures associated with iris dilation and constriction, which provides valuable insight into human iris physiology. Van Zyl et al. created a more extensive snRNA-seq atlas of 191,992 single nuclei across six healthy anterior segment tissues and identified more than 60 cell types [43]. Notably, they demonstrated cell-type specific expression of 924 genes implicated in various ocular pathology susceptibility. For instance, they found that genes implicated in glaucoma and anterior segment dysgenesis (*PAX6* (paired box Pax-6), *PITX2* (paired like homeodomain 2)*, FOXC1, CYP1B1, LTBP2* (latent transforming growth factor beta binding protein 2)*, FOXE3* (forkhead box E3)*, PITX3* (paired-like homeodomain transcription factor 3*), B3GLCT* (beta 3-glucosyltransferase*), COL4A1 (*collagen alpha-1(IV*)), PXDN (*peroxidasin*)*, and *CPAMD8* (C3 and PZP-like alpha-2-macroglobulin domain-containing protein 8)) continued to be highly expressed in adult anterior segment cell types. Genes implicated in ectopia lentis pathogenesis were found to be expressed in non-pigmented ciliary epithelium and transitional lens epithelial cells. *LOXL1* (lysyl oxidase like 1), which is implicated in pseudoexfoliation syndrome, was highly expressed in equatorial lens epithelium. These data can be used to validate disease-gene associations and provides more insight into disease pathogenesis and therapeutic strategies.

## Experimental considerations and limitations of scrna-seq in ophthalmology

While there have been significant advances in noise reduction, sensitivity, and throughput of ophthalmological single-cell transcriptomics studies, it is worthwhile to discuss their limitations in order to enable precise interpretation of their data. One notable limitation is dissociation-induced artefactual transcriptional stress responses that can exacerbate technical noise in the data. Sampling a much larger cell number may overcome this bias, but this may not be financially or logistically feasible. Because datasets involving pathological ocular tissue are complicated by disease severity, dissociation procedure standardization should be an area of further research, particularly for rare or diseased ocular tissues [3]. Another scRNA-seq study limitation is gene expression alteration depending on whether the tissue is fresh or frozen [1]. This presents challenges when tissue availability is unpredictable or when biopsy is required. Sample preparation and single cell dissociation methods may result in significant variations in sensitivity and accuracy [89]. Similarly, the extent to which scRNA-seq datasets of in vitro models correspond with in vivo conditions in humans is unclear.

Additionally, all scRNA-seq protocols to date require several nanograms of RNA as starting material, which renders RNA depletion challenging [14]. Because it is not yet feasible to sequence RNA directly from single cells, all existing scRNA-seq methods are DNA-dependent. The challenge of converting RNA to cDNA lies in minimizing RNA losses, preserving RNA integrity, and accounting for quantitative biases or distortions resulting from cDNA amplification. PCR amplification used for full-length methods can cause significant amplification noise [14]. 3’ amplification bias can result from protocols that selectively sequence partial transcripts at the 3’ end. UMIs can reduce amplification bias, but these are typically used in protocols only involving the 3’ ends of transcripts. In vitro transcription (IVT) can also potentially reduce amplification-associated noise [14]. Another reverse transcription limitation is related to the mRNA dropout rate, with Haque et al. stating that the mRNA to cDNA conversion rate is as low as 10-40% [16]. All RNA amplification except for tRNA and rRNA is made possible by employing an olig-dT primer that is designed to capture polyadenylated RNA, such as mRNA and some lncRNA. However, this potentially results in loss of other useful non-polyadenylated RNA such as microRNA (miRNA) and piwi-interacting RNA (piRNA) [90].

In comparison to bulk RNA sequencing, the proportion of zero counts is much higher in scRNA-seq, with low abundance transcripts exhibiting a higher probability of going uncaptured by existing scRNA-seq protocols. Increasing sequencing depth is a potential strategy to circumvent this technical limitation. However, studies have shown that most scRNA-seq protocols are saturated at 1 million reads [16]. Another potential strategy is to increase the number of cells sequenced, thereby increasing coverage. Townes et al. proposed using principal component analysis on non-normalized counts [91]. Microfluidic-droplet based technologies like the 10X Genomics platform are high-throughput, but can only allow capture of cells that are less than 35 µm [31]. The capturing rate is also 50–60% in comparison to 70–80% of image-based single-cell platforms like CellenONE X1 (Scienion) that select cells based on cell size (3-500 µm), cell morphology, and the presence of fluorescence markers for sample processing [31]. The 10X genomics platform also does not possess the capacity to perform quality control on single cells based on imaging features such as the ICELL8 Single-Cell System (Takara Bio).

Finally, a notable challenge for computational analysis is the lack of standardization of available bioinformatics tools due to the field’s relative novelty. As of 2019, there are 385 scRNA-seq data analysis tools available, but many of them use either R or Python, programming languages that are challenging to learn for scientists with a non-computational background [92]. While cross-environmental support is rapidly growing, tool selection depends largely on the programming language in question. In recent years, web-based tools like CellxGene (https://cellxgene.cziscience.com/) have become available to facilitate data analysis and visualization for ophthalmologists who have minimal computational experience.

## Conclusions and future directions

This review summarizes recent research findings concerning the application of scRNA-seq in elucidating the ocular anterior segment’s transcriptional heterogeneity and diversity of cellular phenotypes. Their limitations notwithstanding, scRNA-seq technologies harbour unprecedented sensitivity, accuracy, and throughput and have been utilized to elucidate rare cell types, cell-cell interactions, developmental pathways, and intratumor heterogeneity that contribute to anterior segment-related disease pathogenesis. These technologies can potentiate novel therapeutic development and can further enhance our understanding of drug resistance and relapse. Importantly, this technology has provided remarkable insight into limbal stem and progenitor cell populations, information that is essential for advancing corneal regenerative and cell replacement therapies.

With these advances in mind, the ophthalmologist can gain unprecedented insight into diseases affecting the ocular surface. While scRNA-seq is not currently utilized for the purpose of ocular diagnostics, we believe that there is potential to use such data to create molecular diagnostic screening panels based on a select number of biomarkers in a disease state [93, 94], particularly for diseases that lack animal models, such as keratoconus. As described earlier, Wang et al.’s identification of loss of *NEAT1* function in corneal endothelial cells in FECD could serve as an early-stage diagnostic biomarker in the disease process [72]. Similarly, Dou et al.’s identification of *YAP1* and *TEAD1* as key regulators of keratoconus progression in corneal stromal cells could also yield promising advances for the early diagnosis and treatment of keratoconus [26]. The main caveats with harnessing scRNA-seq for diagnostic purposes lies in its prohibitively high cost [95], varying transcript detection sensitivity particularly for transcripts that are expressed at a low level, and high coverage sequencing (0.5–3 million reads per cell) in order to ensure accurate gene expression quantification per cell [96].

Additionally, we anticipate that advances in spatially resolved transcriptomics will enhance scRNA-seq studies by providing structural information regarding differential gene expression and enriched pathways. Integration of ocular cell atlases with those of other organs may produce unified insights of systemic diseases, such as in uveitis induced by HLA-B27-associated inflammatory spondyloarthropathies or in orbital metastasis from various cancers [97]. ScRNA-seq harbours enormous potential for elucidating the evolution of cell-cell interactions and gene expression in intraocular and orbital inflammation. Unbiased classification of large numbers of single nuclei in the ocular anterior segment can be further refined by applying DroNc-seq, a novel snRNA-seq method developed by Habib et al. that combines Drop-seq and snRNA-seq [98]. Finally, scRNA-seq applications can be further enhanced by enabling integrated fluorescence detection, separation of cells based on larger cell sizes from 3 to 500 μm and cell morphology, and full length transcript coverage instead of just 3ʹ or 5ʹ fragments [31]. In the coming years, we believe that scRNA-seq integration with clinical data will further enhance our understanding of ocular pathogenesis and help identify novel therapeutic targets [3].

## Supplementary information

Supplementary methods

## References

[1] Voigt AP, Mullin NK, Stone EM, Tucker BA, Scheetz TE, Mullins RF (2021). Single-cell RNA sequencing in vision research: Insights into human retinal health and disease. Prog Retin Eye Res.

[2] Owen N, Moosajee M (2019). RNA-sequencing in ophthalmology research: considerations for experimental design and analysis. Ther Adv Ophthalmol.

[3] Rossin EJ, Sobrin L, Kim LA (2021). Single-cell RNA sequencing: An overview for the ophthalmologist. Semin Ophthalmol.

[4] Eagle RC (2008). Immunohistochemistry in diagnostic ophthalmic pathology: a review. Clin Exp Ophthalmol.

[5] Byers RJ, Hoyland JA, Dixon J, Freemont AJ (2000). Subtractive hybridization—genetic takeaways and the search for meaning. Int J Exp Pathol.

[6] den Hollander AI, van Driel MA, de Kok YJ, van de Pol DJ, Hoyng CB, Brunner HG (1999). Isolation and mapping of novel candidate genes for retinal disorders using suppression subtractive hybridization. Genomics.

[7] Kubota R, McGuire C, Dierks B, Reh TA (2004). Identification of ciliary epithelial-specific genes using subtractive libraries and cDNA arrays in the avian eye. Dev Dyn.

[8] Sharma S, Chang JT, Della NG, Campochiaro PA, Zack DJ (2002). Identification of novel bovine RPE and retinal genes by subtractive hybridization. Mol Vis.

[9] Walker TM, van Ginkel PR, Gee RL, Ahmadi H, Subramanian L, Ksander BR (2002). Expression of angiogenic factors Cyr61 and tissue factor in uveal melanoma. Arch Ophthalmol.

[10] Liu F, Jenssen TK, Trimarchi J, Punzo C, Cepko CL, Ohno-Machado L (2007). Comparison of hybridization-based and sequencing-based gene expression technologies on biological replicates. BMC Genomics.

[11] Trapnell C, Williams BA, Pertea G, Mortazavi A, Kwan G, van Baren MJ (2010). Transcript assembly and quantification by RNA-Seq reveals unannotated transcripts and isoform switching during cell differentiation. Nat Biotechnol.

[12] Hutchins AP, Poulain S, Fujii H, Miranda-Saavedra D (2012). Discovery and characterization of new transcripts from RNA-seq data in mouse CD4(+) T cells. Genomics.

[13] Halperin RF, Hegde A, Lang JD, Raupach EA, Legendre C, C4RCD Research Group (2021). Improved methods for RNAseq-based alternative splicing analysis. Sci Rep..

[14] Zerti D, Collin J, Queen R, Cockell SJ, Lako M (2020). Understanding the complexity of retina and pluripotent stem cell derived retinal organoids with single cell RNA sequencing: current progress, remaining challenges and future prospective. Curr Eye Res.

[15] Tang F, Barbacioru C, Wang Y, Nordman E, Lee C, Xu N (2009). mRNA-Seq whole-transcriptome analysis of a single cell. Nat Methods.

[16] Haque A, Engel J, Teichmann SA, Lönnberg T (2017). A practical guide to single-cell RNA-sequencing for biomedical research and clinical applications. Genome Med.

[17] Ying P, Huang C, Wang Y, Guo X, Cao Y, Zhang Y (2021). Single-cell RNA sequencing of retina:new looks for gene marker and old diseases. Front Mol Biosci.

[18] Gautam P, Hamashima K, Chen Y, Zeng Y, Makovoz B, Parikh BH (2021). Multi-species single-cell transcriptomic analysis of ocular compartment regulons. Nat Commun.

[19] Collin J, Queen R, Zerti D, Bojic S, Dorgau B, Moyse N (2021). A single cell atlas of human cornea that defines its development, limbal progenitor cells and their interactions with the immune cells. Ocul Surf.

[20] Kaplan N, Wang J, Wray B, Patel P, Yang W, Peng H (2019). Single-cell RNA transcriptome helps define the limbal/corneal epithelial stem/early transit amplifying cells and how autophagy affects this population. Invest Ophthalmol Vis Sci.

[21] Wang J, Rattner A, Nathans J (2021). A transcriptome atlas of the mouse iris at single-cell resolution defines cell types and the genomic response to pupil dilation. Elife.

[22] Ligocki AJ, Fury W, Gutierrez C, Adler C, Yang T, Ni M (2021). Molecular characteristics and spatial distribution of adult human corneal cell subtypes. Sci Rep..

[23] Patel G, Fury W, Yang H, Gomez-Caraballo M, Bai Y, Yang T (2020). Molecular taxonomy of human ocular outflow tissues defined by single-cell transcriptomics. Proc Natl Acad Sci USA.

[24] Altshuler A, Amitai-Lange A, Tarazi N, Dey S, Strinkovsky L, Hadad-Porat S (2021). Discrete limbal epithelial stem cell populations mediate corneal homeostasis and wound healing. Cell Stem Cell.

[25] Li DQ, Kim S, Li JM, Gao Q, Choi J, Bian F (2021). Single-cell transcriptomics identifies limbal stem cell population and cell types mapping its differentiation trajectory in limbal basal epithelium of human cornea. Ocul Surf.

[26] Dou S, Wang Q, Zhang B, Wei C, Wang H, Liu T (2022). Single-cell atlas of keratoconus corneas revealed aberrant transcriptional signatures and implicated mechanical stretch as a trigger for keratoconus pathogenesis. Cell Disco.

[27] Fuellen G, Jünemann A (2022). Gene expression data for investigating glaucoma treatment options and pharmacology in the anterior segment, state-of-the-art and future directions. Front Neurosci.

[28] Wu H, Kirita Y, Donnelly EL, Humphreys BD (2019). Advantages of single-nucleus over single-cell RNA sequencing of adult kidney: rare cell types and novel cell states revealed in fibrosis. J Am Soc Nephrol.

[29] Malik KJ, Chen CD, Olsen TW (2003). Stability of RNA from the retina and retinal pigment epithelium in a porcine model simulating human eye bank conditions. Invest Ophthalmol Vis Sci.

[30] Kallestad L, Blackshaw S, Khalil AM, Palczewski K (2019). Tissue- and species-specific patterns of RNA metabolism in post-mortem mammalian retina and retinal pigment epithelium. Sci Rep..

[31] Shomroni O, Sitte M, Schmidt J, Parbin S, Ludewig F, Yigit G (2022). A novel single-cell RNA-sequencing approach and its applicability connecting genotype to phenotype in ageing disease. Sci Rep..

[32] Hwang B, Lee JH, Bang D (2018). Single-cell RNA sequencing technologies and bioinformatics pipelines. Exp Mol Med.

[33] Picelli S (2017). Single-cell RNA-sequencing: the future of genome biology is now. RNA Biol.

[34] Svensson V, Natarajan KN, Ly LH, Miragaia RJ, Labalette C, Macaulay IC (2017). Power analysis of single-cell RNA-sequencing experiments. Nat Methods.

[35] Bacher R, Kendziorski C (2016). Design and computational analysis of single-cell RNA-sequencing experiments. Genome Biol.

[36] Ziegenhain C, Vieth B, Parekh S, Reinius B, Guillaumet-Adkins A, Smets M (2017). Comparative analysis of single-cell RNA sequencing methods. Mol Cell.

[37] Stuart T, Butler A, Hoffman P, Hafemeister C, Papalexi E, Mauck WM (2019). Comprehensive integration of single-cell data. Cell..

[38] McCarthy DJ, Campbell KR, Lun ATL, Wills QF (2017). Scater: pre-processing, quality control, normalization and visualization of single-cell RNA-seq data in R. Bioinformatics..

[39] Wolf FA, Angerer P, Theis FJ (2018). SCANPY: large-scale single-cell gene expression data analysis. Genome Biol.

[40] 1. Davis S KRZLSJKV olivier PBOKKGACK et al. seandavi/awesome-single-cell. 2018.

[41] Butler A, Hoffman P, Smibert P, Papalexi E, Satija R (2018). Integrating single-cell transcriptomic data across different conditions, technologies, and species. Nat Biotechnol.

[42] Ronan T, Qi Z, Naegle KM (2016). Avoiding common pitfalls when clustering biological data. Sci Signal.

[43] van Zyl T, Yan W, McAdams AM, Monavarfeshani A, Hageman GS, Sanes JR (2022). Cell atlas of the human ocular anterior segment: tissue-specific and shared cell types. Proc Natl Acad Sci USA.

[44] van Zyl T, Yan W, McAdams A, Peng YR, Shekhar K, Regev A (2020). Cell atlas of aqueous humor outflow pathways in eyes of humans and four model species provides insight into glaucoma pathogenesis. Proc Natl Acad Sci USA.

[45] Sun C, Wang H, Ma Q, Chen C, Yue J, Li B (2021). Time-course single-cell RNA sequencing reveals transcriptional dynamics and heterogeneity of limbal stem cells derived from human pluripotent stem cells. Cell Biosci.

[46] Alam J, Yazdanpanah G, Ratnapriya R, Borcherding N, de Paiva CS, Li D (2022). Single-cell transcriptional profiling of murine conjunctival immune cells reveals distinct populations expressing homeostatic and regulatory genes. Mucosal Immunol.

[47] Alam J, Yazdanpanah G, Ratnapriya R, Borcherding N, de Paiva CS, Li D (2022). IL-17 producing lymphocytes cause dry eye and corneal disease with aging in RXRα mutant mouse. Front Med (Lausanne).

[48] Hodge RD, Bakken TE, Miller JA, Smith KA, Barkan ER, Graybuck LT (2019). Conserved cell types with divergent features in human versus mouse cortex. Nature.

[49] Hoffmann M, Kleine-Weber H, Schroeder S, Krüger N, Herrler T, Erichsen S (2020). SARS-CoV-2 cell entry depends on ACE2 and TMPRSS2 and is blocked by a clinically proven protease inhibitor. Cell.

[50] Ziegler CGK, Allon SJ, Nyquist SK, Mbano IM, Miao VN, Tzouanas CN (2020). SARS-CoV-2 receptor ACE2 is an interferon-stimulated gene in human airway epithelial cells and is detected in specific cell subsets across tissues. Cell.

[51] Bugge TH, Antalis TM, Wu Q (2009). Type II transmembrane serine proteases. J Biol Chem.

[52] Wu P, Duan F, Luo C, Liu Q, Qu X, Liang L (2020). Characteristics of ocular findings of patients with coronavirus disease 2019 (COVID-19) in Hubei Province, China. JAMA Ophthalmol.

[53] Collin J, Queen R, Zerti D, Dorgau B, Georgiou M, Djidrovski I (2021). Co-expression of SARS-CoV-2 entry genes in the superficial adult human conjunctival, limbal and corneal epithelium suggests an additional route of entry via the ocular surface. Ocul Surf.

[54] Ma R, Gan L, Jiang S, Ding P, Chen D, Wu J (2021). High expression of SARS-CoV-2 entry factors in human conjunctival goblet cells. Exp Eye Res.

[55] Jackson RM, Hatton CF, Spegarova JS, Georgiou M, Collin J, Stephenson E (2022). Conjunctival epithelial cells resist productive SARS-CoV-2 infection. Stem Cell Rep..

[56] Armstrong L, Collin J, Mostafa I, Queen R, Figueiredo FC, Lako M (2021). In the eye of the storm: SARS-CoV-2 infection and replication at the ocular surface?. Stem Cells Transl Med.

[57] Lou B, Zeng L, Gao X, Qian X, Li JJ, Gu X (2022). A single-cell transcriptomic atlas of the human ciliary body. Cell Mol Life Sci.

[58] Youkilis JC, Bassnett S (2021). Single-cell RNA-sequencing analysis of the ciliary epithelium and contiguous tissues in the mouse eye. Exp Eye Res.

[59] Farnsworth DR, Posner M, Miller AC (2021). Single cell transcriptomics of the developing zebrafish lens and identification of putative controllers of lens development. Exp Eye Res.

[60] Grifone R, Saquet A, Xu Z, Shi DL (2018). Expression patterns of Rbm24 in lens, nasal epithelium, and inner ear during mouse embryonic development. Dev Dyn.

[61] Siddam AD, Gautier-Courteille C, Perez-Campos L, Anand D, Kakrana A, Dang CA (2018). The RNA-binding protein Celf1 post-transcriptionally regulates p27Kip1 and Dnase2b to control fiber cell nuclear degradation in lens development. PLoS Genet.

[62] Kameishi S, Umemoto T, Matsuzaki Y, Fujita M, Okano T, Kato T (2016). Characterization of rabbit limbal epithelial side population cells using RNA sequencing and single-cell qRT-PCR. Biochem Biophys Res Commun.

[63] He J, Ou S, Ren J, Sun H, He X, Zhao Z (2020). Tissue engineered corneal epithelium derived from clinical-grade human embryonic stem cells. Ocul Surf.

[64] Dou S, Wang Q, Qi X, Zhang B, Jiang H, Chen S (2021). Molecular identity of human limbal heterogeneity involved in corneal homeostasis and privilege. Ocul Surf.

[65] Lavker RM, Kaplan N, Wang J, Peng H (2020). Corneal epithelial biology: Lessons stemming from old to new. Exp Eye Res.

[66] Català P, Groen N, Dehnen JA, Soares E, van Velthoven AJH, Nuijts RMMA (2021). Single cell transcriptomics reveals the heterogeneity of the human cornea to identify novel markers of the limbus and stroma. Sci Rep..

[67] Li JM, Kim S, Zhang Y, Bian F, Hu J, Lu R (2021). Single-cell transcriptomics identifies a unique entity and signature markers of transit-amplifying cells in human corneal limbus. Invest Ophthalmol Vis Sci.

[68] Li Y, Jeong J, Song W (2022). Molecular characteristics and distribution of adult human corneal immune cell types. Front Immunol.

[69] Eriksen AZ, Møller R, Makovoz B, Uhl SA, tenOever BR, Blenkinsop TA (2021). SARS-CoV-2 infects human adult donor eyes and hESC-derived ocular epithelium. Cell Stem Cell.

[70] Wieghofer P, Hagemeyer N, Sankowski R, Schlecht A, Staszewski O, Amann L (2021). Mapping the origin and fate of myeloid cells in distinct compartments of the eye by single-cell profiling. EMBO J.

[71] Bargagna-Mohan P, Schultz G, Rheaume B, Trakhtenberg EF, Robson P, Pal-Ghosh S (2021). Corneal nonmyelinating Schwann cells illuminated by single-cell transcriptomics and visualized by protein biomarkers. J Neurosci Res.

[72] Wang Q, Dou S, Zhang B, Jiang H, Qi X, Duan H (2022). Heterogeneity of human corneal endothelium implicates lncRNA NEAT1 in Fuchs endothelial corneal dystrophy. Mol Ther Nucleic Acids.

[73] Burkholder BM, Jabs DA (2021). Uveitis for the non-ophthalmologist. BMJ..

[74] Tsirouki T, Dastiridou A, Symeonidis C, Tounakaki O, Brazitikou I, Kalogeropoulos C (2018). A focus on the epidemiology of uveitis. Ocul Immunol Inflamm.

[75] Kasper M, Heming M, Schafflick D, Li X, Lautwein T, Horste MMZ (2021). Intraocular dendritic cells characterize HLA-B27-associated acute anterior uveitis. Elife..

[76] Hassman LM, Paley MA, Esaulova E, Paley GL, Ruzycki PA, Linskey N (2021). Clinicomolecular identification of conserved and individualized features of granulomatous uveitis. Ophthalmol Sci.

[77] Thomson BR, Liu P, Onay T, Du J, Tompson SW, Misener S (2021). Cellular crosstalk regulates the aqueous humor outflow pathway and provides new targets for glaucoma therapies. Nat Commun.

[78] Thomson BR, Quaggin SE (2022). Preparation of a single cell suspension from the murine iridocorneal angle. Bio Protoc.

[79] van Buskirk EM (1989). The anatomy of the limbus. Eye (Lond).

[80] Wu H, Chen W, Zhao F, Zhou Q, Reinach PS, Deng L (2018). Scleral hypoxia is a target for myopia control. Proc Natl Acad Sci USA.

[81] Chattopadhyay C, Kim DW, Gombos DS, Oba J, Qin Y, Williams MD (2016). Uveal melanoma: From diagnosis to treatment and the science in between. Cancer..

[82] Pandiani C, Strub T, Nottet N, Cheli Y, Gambi G, Bille K (2021). Single-cell RNA sequencing reveals intratumoral heterogeneity in primary uveal melanomas and identifies HES6 as a driver of the metastatic disease. Cell Death Differ.

[83] Strub T, Martel A, Nahon-Esteve S, Baillif S, Ballotti R, Bertolotto C (2021). Translation of single-cell transcriptomic analysis of uveal melanomas to clinical oncology. Prog Retin Eye Res.

[84] Gao G, Deng A, Liang S, Liu S, Fu X, Zhao X (2022). Integration of bulk RNA sequencing and single-cell RNA sequencing to reveal uveal melanoma tumor heterogeneity and cells related to survival. Front Immunol.

[85] Durante MA, Rodriguez DA, Kurtenbach S, Kuznetsov JN, Sanchez MI, Decatur CL (2020). Single-cell analysis reveals new evolutionary complexity in uveal melanoma. Nat Commun.

[86] Bakhoum MF, Francis JH, Agustinus A, Earlie EM, di Bona M, Abramson DH (2021). Loss of polycomb repressive complex 1 activity and chromosomal instability drive uveal melanoma progression. Nat Commun.

[87] Bigot J, Lalanne AI, Lucibello F, Gueguen P, Houy A, Dayot S (2021). Splicing patterns in SF3B1-mutated uveal melanoma generate shared immunogenic tumor-specific neoepitopes. Cancer Disco.

[88] Liang Q, Dharmat R, Owen L, Shakoor A, Li Y, Kim S (2019). Single-nuclei RNA-seq on human retinal tissue provides improved transcriptome profiling. Nat Commun.

[89] Wagner A, Regev A, Yosef N (2016). Revealing the vectors of cellular identity with single-cell genomics. Nat Biotechnol.

[90] Yang Q, Li R, Lyu Q, Hou L, Liu Z, Sun Q (2019). Single-cell CAS-seq reveals a class of short PIWI-interacting RNAs in human oocytes. Nat Commun.

[91] Townes FW, Hicks SC, Aryee MJ, Irizarry RA (2019). Feature selection and dimension reduction for single-cell RNA-Seq based on a multinomial model. Genome Biol.

[92] Zappia L, Phipson B, Oshlack A (2018). Exploring the single-cell RNA-seq analysis landscape with the scRNA-tools database. PLoS Comput Biol.

[93] Kim N, Eum HH, Lee HO (2021). Clinical perspectives of single-cell RNA sequencing. Biomolecules.

[94] Kim J, Xu Z, Marignani PA (2021). Single-cell RNA sequencing for the identification of early-stage lung cancer biomarkers from circulating blood. NPJ Genom Med.

[95] Ding S, Chen X, Shen K (2020). Single-cell RNA sequencing in breast cancer: Understanding tumor heterogeneity and paving roads to individualized therapy. Cancer Commun (Lond).

[96] Mandric I, Schwarz T, Majumdar A, Hou K, Briscoe L, Perez R (2020). Optimized design of single-cell RNA sequencing experiments for cell-type-specific eQTL analysis. Nat Commun.

[97] Eraslan G, Drokhlyansky E, Anand S, Fiskin E, Subramanian A, Slyper M (2022). Single-nucleus cross-tissue molecular reference maps toward understanding disease gene function. Science.

[98] Habib N, Avraham-Davidi I, Basu A, Burks T, Shekhar K, Hofree M (2017). Massively parallel single-nucleus RNA-seq with DroNc-seq. Nat Methods.

