# Supplementary material for: Single-cell transcriptomics of the ocular anterior segment: a comprehensive review
Source: Eye (Lond). 2023 May 3;37(16):3334–50. doi: 10.1038/s41433-023-02539-3 (PMC10156079; doi:10.1038/s41433-023-02539-3)
Supplement: Supplementary file 1 — Supplementary methods [file 41433_2023_2539_MOESM1_ESM.docx]

**Supplementary Information**

Additional details describing the literature search algorithm used to identify relevant publications for this review article.

**Methods**

The keywords listed in the Methods section were divided into three groups. Group 1 terms were anatomical structure-related and included ‘single-cell RNA seq cornea’ OR ‘single-cell RNA seq lens’ OR ‘single-cell RNA seq aqueous humor’ OR ‘single nuclei RNA seq anterior segment’ OR ‘single cell RNA seq anterior segment.’ ‘ScRNA-seq’ terms with the corresponding anatomical structures were also utilized to ensure that no studies were missed. Group 2 terms were condition-related and included ‘single cell RNA seq COVID-19’ OR ‘single cell RNA cataract’ OR ‘single-cell RNA glaucoma’ OR ‘single cell RNA uveal melanoma.’ Group 3 terms were human vs. animal model-related and included ‘single cell RNA seq human eye’ OR ‘single cell RNA seq murine eye’ OR ‘single cell RNA sequencing animal models’ OR ‘single cell RNA sequencing organoids’.

The literature search was performed using the following algorithm: search for any terms in group 1 (anatomical structure-related) in combination with each of the terms in group 2 (condition-related) or group 3 (model-related).
